# New estimates and synthesis of chromosome numbers, ploidy levels and genome size variation in *Allium* sect. *Codonoprasum*: advancing our understanding of the unresolved diversification and evolution of this section

**DOI:** 10.1186/s40529-024-00446-8

**Published:** 2024-12-24

**Authors:** Lucie Kobrlová, Michaela Jandová, Kateřina Vojtěchová, Lenka Šafářová, Martin Duchoslav

**Affiliations:** 1 Plant Biosystematics and Ecology RG, Department of Botany, Faculty of Science, Palacky University, Šlechtitelů 11, 779 00 Olomouc, Czech Republic; 2 Institute of Botany, Czech Academy of Sciences, Zámek 1, 252 43 Průhonice, Czech Republic; 3 East Bohemian Museum, Zámek 2, 530 02 Pardubice, Czech Republic

**Keywords:** Chromosome number, Cytogeography, DNA ploidy level, Flow cytometry, Genome size, Polyploidy

## Abstract

**Background:**

The genus *Allium* is known for its high chromosomal variability, but most chromosome counts are based on a few individuals and genome size (GS) reports are limited in certain taxonomic groups. This is evident in the *Allium* sect. *Codonoprasum*, a species-rich (> 150 species) and taxonomically complex section with weak morphological differences between taxa, the presence of polyploidy and frequent misidentification of taxa. Consequently, a significant proportion of older karyological reports may be unreliable and GS data are lacking for the majority of species within the section. This study, using chromosome counting and flow cytometry (FCM), provides the first comprehensive and detailed insight into variation in chromosome number, polyploid frequency and distribution, and GS in section members, marking a step towards understanding the unresolved diversification and evolution of this group.

**Results:**

We analysed 1578 individuals from 316 populations of 25 taxa and reported DNA ploidy levels and their GS, with calibration from chromosome counts in 22 taxa. Five taxa had multiple ploidy levels. First estimates of GS were obtained for 16 taxa. A comprehensive review of chromosome number and DNA-ploidy levels in 129 taxa of the section revealed that all taxa have *x* = 8, except *A. rupestre* with two polyploid series (*x* = 8, descending dysploidy *x* = 7), unique for this section. Diploid taxa dominated (72.1%), while di- & polyploid (12.4%) and exclusively polyploid (15.5%) taxa were less common. Ploidy diversity showed that diploid taxa dominated in the eastern Mediterranean and decreased towards the west and north, whereas only polyploid cytotypes of di- & polyploid taxa or exclusively polyploid taxa dominated in northern and northwestern Europe. A 4.1-fold variation in GS was observed across 33 taxa analysed so far (2C = 22.3–92.1 pg), mainly due to polyploidy, with GS downsizing observed in taxa with multiple ploidy levels. Intra-sectional GS variation suggests evolutionary relationships, and intraspecific GS variation within some taxa may indicate taxonomic heterogeneity and/or historical migration patterns.

**Conclusions:**

Our study showed advantages of FCM as an effective tool for detecting ploidy levels and determining GS within the section. GS could be an additional character in understanding evolution and phylogenetic relationships within the section.

**Supplementary Information:**

The online version contains supplementary material available at 10.1186/s40529-024-00446-8.

## Background

As the evolutionary history of organisms is inscribed in their chromosomes, chromosome number is a fundamental genomic attribute of an organism (Mayrose and Lysák 2020). Information on chromosome number and nuclear DNA content highlights the role of numerical variation (Greilhuber et al. 2005; Rice et al. 2015; Pellicer and Leitch 2020; Siljak-Yakovlev et al. 2020) and, together with the study of karyotypic differentiation, helps to understand the role of structural changes in evolutionary processes (e.g., Schubert and Lysák 2011; Mandáková and Lysák 2018). Chromosome number and genome size (hereafter GS) are important species-specific traits (Stebbins 1971; Guerra 2008, 2012; Goldblatt and Lowry 2011; Carta et al. 2020; Pellicer and Leitch 2020), which are useful tools for discriminating between taxa and resolving taxonomy within groups that are critical, for example, due to morphological reduction, phenotypic plasticity, mating systems and reticulate evolution (e.g., Castro et al. 2012; Hajrudinović et al. 2015; Prančl et al. 2018; Popelka et al. 2019; Afonso et al. 2021). GS and its intraspecific variation may also help to understand the evolutionary forces shaping genomic features (Šmarda and Bureš 2010; Prančl et al. 2014, 2018; Becher et al. 2021) and functional diversity of plants (e.g., Šímová and Herben 2012; Roddy et al. 2020; Bitomský et al. 2022, 2023).

Reports on the chromosome numbers are usually based on small numbers of individuals. Such an approach may underestimate the variability in cytotype composition within and between populations, which is usually the result of genome duplication (Stuessy 2009). From a practical perspective, flow cytometry has proven valuable in plant biosystematics over the last two decades (Bourge et al. 2018; Sliwinska et al. 2022; Loureiro et al. 2023). This technique allows rapid and non-destructive estimation of DNA-ploidy levels and nuclear DNA content in a large number of samples (e.g., Trávníček et al. 2012; Čertner et al. 2017, 2022; Rejlová et al. 2019). Its application has led to the discovery of diverse cytotypes in various plant taxa, providing a better understanding of the mechanisms underlying cytotype origin and coexistence (reviewed in Kolář et al. 2017). Additionally, it has shed light on cytogeographic diversity across species ranges (e.g., Mráz et al. 2008; Duchoslav et al. 2010, 2020; Šafářová and Duchoslav 2010; Šafářová et al. 2011; Kobrlová et al. 2016, 2022; Taraška et al. 2021, 2024; Horák et al. 2023; Vejvodová et al. 2024) and has helped to identify patterns of ecological diversification or habitat shifts (e.g., Duchoslav et al. 2020; Kobrlová et al. 2022; Kúr et al. 2023), which can provide insights into the evolutionary history of species (Blommaert 2020; Cang et al. 2024).

The genus *Allium* L. (Amaryllidaceae, Allieae) is one of the largest monocotyledonous genera (Chase et al. 2009; Costa et al. 2020), with over a thousand accepted species (POWO 2024), represented by perennial rhizomatous or bulbiferous herbs that combine sexual and asexual reproduction (Rabinowitch and Currah 2002), and is widely distributed throughout the Northern Hemisphere (Fritsch and Friesen 2002; Friesen et al. 2006; Li et al. 2010; Hauenschild et al. 2017). The genus exhibits multiple basic chromosome numbers, including *x* = 7, 8, 9, 10, 11 (Hanelt et al. 1992; Friesen et al. 2006; Li et al. 2016; Peruzzi et al. 2017; Han et al. 2020). The genus also displays high levels of polyploidy (Friesen 1992; Hanelt et al. 1992; Han et al. 2020) and occasional occurrences of B chromosomes (Speta 1984; Holmes and Bougourd 1989; Vujošević et al. 2013). Polyploidisation is considered the important driver of adaptation and speciation across diverse environments within the genus (Han et al. 2020).

A number of studies have been conducted on the diversity of chromosome number (for a comprehensive survey see Peruzzi et al. 2017; Han et al. 2020) and GS (Leitch et al. 2019) in the genus *Allium*. However, despite the genus's taxonomic richness, certain groups within it are underrepresented in chromosome count and GS databases. This is particularly evident in the *Allium* sect. *Codonoprasum* Rchb. This section is one of the largest and taxonomically most complicated within the genus (Hanelt 1996; Salmeri et al. 2014, 2016; Özhatay and Koçyiğit 2019), exhibiting minor morphological differences between taxa and presence of polyploid species/species groups (Tzanoudakis and Vosa 1988; Peruzzi et al. 2017; Duchoslav et al. 2020; Han et al. 2020). This often leads to misidentification of taxa (Salmeri et al. 2016; Vojtěchová et al. 2023), and thus a significant proportion of older karyological reports for certain species may not be reliable due to uncertain identification of the studied individuals. In addition, GS data are lacking for the majority of species within the section, with only a small subset of taxa having been studied (Ohri et al. 1998; Baranyi and Greilhuber 1999; Duchoslav et al. 2013; Šmarda et al. 2019; Vojtěchová et al. 2023, 2024). Furthermore, more detailed analyses focusing on population-level differences in GS and ploidy composition (i.e., global and local distribution patterns of cytotypes) are almost absent for section members (but see Duchoslav et al. 2010, 2013, 2020; Šafářová and Duchoslav 2010; Šafářová et al. 2011).

To address these issues in a broader context, we collected population samples of 25 taxa (species, subspecies) of *A*. sect. *Codonoprasum* across Europe and neighbouring regions to cover as much of the taxonomic diversity and geographic range of the section as possible. Using classical karyology, flow cytometry and a comprehensive review of the available literature, our aims were to (i) determine the diversity of DNA ploidy levels (sensu Suda et al. 2006) within and between populations for each taxon studied, (ii) validate the detected DNA ploidy levels by chromosome counting, (iii) estimate the GS of the detected ploidy levels and evaluate its variation and spatial pattern, and (iv) critically compare the new data with those from the literature. Finally, we aimed to discuss the patterns obtained in more general content. Therefore, we extracted all available data on chromosome number and GS for the remaining section members not covered by our present research and synthesised the current knowledge on cytogenetic diversity within the section. Our goal is to highlight problematic groups and to stimulate further studies aimed primarily at understanding the taxonomic relationships and phylogeny of this evolutionarily young group.

## Material and methods

### Plant material and species identification

Taxa identification was based on the original species/subspecies descriptions, regional floras, and studies dealing with their taxonomy. Specifically, we largely accepted the most recent treatments of the respective species. Plant individuals were collected between 2004 and 2024 from natural populations across Europe, Caucasus and Israel, with emphasis to cover as much of the range of the taxa studied as possible (Table S1). Plants were transported and cultivated in the experimental garden of Palacký University in Olomouc, Czech Republic, where all analyses were conducted on the cultivated plants. The voucher specimens were deposited in the Herbarium of Palacký University in Olomouc (OL).

### Chromosome counts

Actively growing, young roots were harvested from the pot-cultivated plants, pre-treated with 8-hydroxyquinoline (0.002 M) in darkness at room temperature for 4 h, fixed in a cold mixture of ethanol and acetic acid (3:1) overnight and then stored at 4 °C until use. Selected root tips were hydrolysed in 5 N HCl for 25 min, stained with Schiff reagent for 40 min and squashed in 45% acetic acid (Lillie 1951). Preparations were photographed and counted using an Olympus CX-31 light microscope. Usually, at least five metaphases were counted for each individual studied.

### Flow cytometry

Flow cytometry (FCM) was used to estimate the DNA ploidy level (relative genome size, RGS, i.e. ratio of the 2C-peak of the sample to the 2C-peak of the internal standard; Suda et al. 2006) and to determine nuclear DNA content, i.e. the holoploid genome size (absolute genome size, AGS; 2C value sensu Greilhuber et al. 2005) of samples. The methodical recommendations of Sliwinska et al. (2022) were followed. Chromosome counts for selected individuals analysed by FCM served as reference material for the estimates obtained using FCM. The monoploid genome size (1C*x* value sensu Greilhuber et al. 2005) was calculated as the 2C value of the sample divided by its ploidy level. Samples were prepared according to the protocol described by Duchoslav et al. (2010) and stained with propidium iodide (PI) with addition of RNAse (both 50 μg·ml^–1^). The analyses were performed on a Partec PAS (Partec GmbH, Münster, Germany) or BD Accuri C6 (BD Biosciences, San Jose, USA) cytometer. *Secale cereale* L. ‘Daňkovské’ (2C = 16.19 pg; Doležel et al. 1998); *Triticum aestivum* ‘Saxana’ (2C = 34.24 pg; Šafářová and Duchoslav 2010); *Pisum sativum* ‘Ctirad’ (2C = 8.75 pg; Vojtěchová et al. 2023); *Vicia faba* 'Inovec' (2C = 26.81 pg, the value recalculated to the primary standard *S. cereale*) served as internal standards. Where multiple internal standards were used for FCM within a given taxon, the observed values based on the less frequently used standards were recalculated to the dominant standard and these values are presented.

Separate plants or pooled samples of up to four plants per population were measured for the RGS estimation. For each (pooled) sample, fluorescence intensity of usually 3000 particles were recorded for the RGS estimations. If there was a suspicion that there might be more DNA ploidy levels in a pooled sample, each plant from that sample was reanalysed separately. The following measurement strategy was chosen to ensure validity of the AGS estimation: (i) all measurements were made over period when the plants were in an identical phenological phase of development, with young fresh leaves without any symptoms of senescence or pathogen attack (March to May), (ii) at least 5000 nuclei per sample were recorded, (iii) only CV for the G_0_/G_1_ peaks of the standard and *Allium* samples below 5% (6% in some instances) were accepted, (iv) each sample was measured by the same operator at least three different times on different days and mean AGS value was calculated from these three measurements (Doležel et al. 2007).

### Bibliographic review on chromosome counts and GS

An extensive bibliographic review was conducted using the CCDB metadatabase (Rice et al. 2015), the plant C-value database (Leitch et al. 2019), and our additional independent searches. This review provides a detailed list of chromosome numbers and nuclear DNA contents (2C values) for the members of *A.* sect. *Codonoprasum* estimated by different methods (FCM, Feulgen microdensitometry (FEM), Vickers M86 scanning microdensitometry (VIM), Hardie et al. 2002; Doležel et al. 2007). All records were critically reviewed from a taxonomic perspective to ensure accurate determination and nomenclature, based on the original publications, recent taxonomic concepts, and, where possible, direct contact with the authors of the original descriptions. The assignment of the studied taxa to *A*. sect. *Codonoprasum* usually followed the original description or subsequent taxonomic revisions. Recently, Özhatay and Koçyiğit (2019) transferred 23 species, mostly described from Turkey and originally assigned to *A*. sect. *Codonoprasum*, to *A*. sect. *Scorodon* K. Koch. In this review, we present both taxonomic treatments, i.e. one that accepts this sectional reclassification and one that does not.

The majority of extracted chromosome data sets of the studied taxa, where locality information was available, were georeferenced. Distribution maps were created for different ploidy levels, focusing on selected di- & polyploid and exclusively polyploid taxa with a large number of records. Additionally, all available GS estimates for *Allium* (Leitch et al. 2019) were extracted and used as background data to describe the known variation in GS within the genus.

### Data analyses

Frequency of different cytotype compositions of populations was estimated for each studied taxon based on the presence of cytotypes within populations. Frequency of each cytotype within each taxon was based on the total number of FCM-analysed individuals, ignoring their population assignment. Summary statistics of GS parameters were calculated for each studied taxon (ploidy level), based on population-level data. Relationship between genome size (AGS, RGS) and geographic coordinates (latitude, longitude) for selected ploidy levels of the taxa studied was assessed by Spearman correlation coefficient. Data were analysed in NCSS 9 (www.ncss.com). The maps were created in QGIS 3.28 (www.qgis.org), using the Terrain Elevation Above Sea Level map provided by the Global Solar Atlas 2.0 (https://globalsolaratlas.info) as a background.

## Results and discussion

### Chromosome number, DNA-ploidy level and GS assessment for 25 studied taxa

The new data on chromosome numbers, the diversity and frequency of cytotypes in their populations and the RGS and AGS for each ploidy level in each taxon are summarised in Table 1, while Table S1 gives these data for the individual populations studied. Detailed bibliographic reviews of published karyological and GS data for the taxa studied are given in Tables S2 and S3, respectively. A total of 25 taxa were analysed by FCM (316 populations/1578 individuals), with first AGS estimates provided for 16 taxa (Table 1). For nine taxa, several reports on nuclear DNA content have been previously published (Table S3), whereas for three of them we detected the presence of new cytotypes. More than one ploidy level was identified in five taxa. Chromosome numbers were counted for 22 taxa, with new reports for *A. rupestre* Steven and *A. dinaricum* Bogdanović et al. Multiple ploidy levels were confirmed in four taxa. A commentary on the data obtained for each taxon studied is provided below, together with critical assessment of the available literature.

**Table 1:** New estimates of chromosome number, DNA-ploidy level (RGS), detected population ploidy composition, estimates of holoploid genome size (AGS, 2C value), monoploid genome size (1C*x*) of the studied *Allium* taxa

Taxon	Npop	Nind (mean)	Ploidy_comp (count, % of Npop)	Ploidy level (2n)	Ploidy level (% of Nind)	Chrom (2n)	Nind (RGS)	RGS (mean)	RGS (SD)	RGS (population min–max)	Var (%)	Npop (AGS)	Nind (AGS)	AGS (mean, pg)	AGS (SD, pg)	AGS (pop. min–max, pg)	CV (%) stand.	CV (%) sample	1C*x *(mean, pg)	Prime AGS	Primary stand.
*A. aethense*	1	6 (6.0)	2*x* (100.0%)	2*x*	100.0	16	6	1.453	0.017	–		1	2	23.5	0.3	–	2.99	3.37	11.8	!,!	*Secale*
*A. carinatum* subsp.* carinatum*	75	414 (5.5)	2*x* (12, 16.0%), 3*x* (58, 77.3%), 4*x* (2, 2.7%), 2*x* + 3*x* (2, 2.7%), 2*x* + 4*x* (1, 1.3%)	2*x*	19.8	16	82	2.074	0.135	1.899–2.280	18.4	9	22	32.9	1.8	31.2–36.9	2.72	2.82	16.5	–,!	*Secale*
				3*x*	77.8	24	322	2.974	0.093	2.777–3.180	13.6	35	95	47.9	1.5	45.0–51.7	3.10	3.09	16.0	–,–	*Secale*
				4*x* (cf.)	2.4	–	10	3.569	0.06	3.505–3.624	3.3	1	1	56.7	–	–	3.13	2.72	14.2	!,!	*Secale*
*A. carinatum* subsp*. pulchellum*	21	120 (5.7)	2*x* (100.0%)	2*x*	100.0	16	120	2.086	0.213	1.740–2.398	31.5	18	46	33.6	3.6	28.1–38.8	3.02	3.04	16.8	–,–	*Secale*
*A. daninianum*	3	10 (3.3)	2*x* (100.0%)	2*x*	100.0	16	10	1.963	0.061	1.953–2.029	3.9	2	2	31.6	0.9	30.9–32.2	2.90	3.31	15.8	!,!	*Secale*
*A. dentiferum*	43	233 (5.4)	4*x* (10, 23.3%), 5*x* (33, 76.7%)	4*x*	28.3	32	66	1.372	0.041	1.272–1.421	10.9	8	8	47.8	0.6	46.6–48.7	2.96	3.36	12.0	!,!	*Triticum*
				5*x*	71.7	40	167	1.662	0.046	1.551–1.754	12.2	17	19	57.3	1.1	55.4–59.7	3.40	3.82	11.5	!,!	*Triticum*
*A. "dentiferum-pallens"*	9	26 (2.9)	4*x* (100.0%)	4*x*	100.0	32	25	1.689	0.100	1.556–1.851	17.4	6	8	45.1	2.8	41.9–49.8	3.49	3.73	11.3	!,!	*Vicia*
*A. dinaricum*	2	13 (6.5)	2*x* (1, 50.0%), 2*x* + 2 (1, 50.0%)	2*x*	84.6	–	11	2.021	0.098	1.952–2.089	6.8	2	2	32.2	0.7	31.7–32.7	2.70	2.95	16.1	!,!	*Secale*
				2*x* + 2	15.4	18	2	2.198	0.024	–	–	1	2	35.6	0.4	–	2.56	2.90	–	!,!	*Secale*
*A. flavum* subsp*. flavum*	76	337 (4.4)	2*x* (68, 89.5%), 2*x* + 4*x* (1, 1.3%), 4*x* (7, 9.2%)	2*x*	89.9	16	303	1.744	0.084	1.320–1.921	34.5	40	67	27.9 (28.1)*	1.8	21.4–31.4	2.79	2.98	14.0	–,–	*Secale*
				4*x*	10.1	32	34	2.667	0.164	2.343–2.807	17.4	7	17	42.9	2.7	38.0–45.5	3.43	3.12	10.7	–,!	*Secale*
*A. flavum* subsp*. tauricum*	19	62 (3.3)	2*x* (5, 26.3%), 4*x* (14, 73.7%)	2*x* (cf.)	32.3	–	20	1.911	0.036	1.858–1.953	5.0	3	13	31.1	0.3	30.9–31.6	2.65	2.70	15.6	!,!	*Secale*
				4*x*	67.7	32	42	2.527	0.168	2.256–2.870	24.3	11	19	41.0	1.9	38.0–44.3	3.33	3.38	10.3	–,!	*Secale*
*A. garbarii*	1	4 (4.0)	2*x* (100.0%)	2*x*	100.0	16	4	1.460	0.005	–	–	1	1	23.7	–	–	3.34	3.50	11.9	!,!	*Secale*
*A. guicciardii*	1	8 (8.0)	2*x* (8, 100.0%)	2*x*	100.0	–	8	1.756	0.064	–	–	1	1	28.2	–	–	2.05	1.94	14.1	!,!	*Secale*
*A. hermoneum*	2	2 (1.0)	4*x* (2, 100.0%)	4*x*	100.0	32	2	1.662	0.067	1.614–1.709	5.7	1	1	58.5	–	–	3.40	4.19	14.6	!,!	*Triticum*
*A. karsianum*	3	5 (1.7)	2*x* (5, 100.0%)	2*x*	100.0	16	5	2.177	0.027	2.151–2.205	2.5	3	3	35.4	0.3	35.2–35.7	2.59	2.59	17.3	–,!	*Secale*
*A. kunthianum*	3	10 (3.3)	2*x* (10, 100.0%)	2*x*	100.0	16	10	1.997	0.018	1.987–2.017	1.5	3	5	32.4	0.3	32.2–32.7	3.60	3.69	16.2	–,!	*Secale*
*A. melanantherum*	2	14 (7.0)	3*x* (100.0%)	3*x*	100.0	24	14	2.487	0.018	2.474–2.499	1.0	2	9	40.3	0.4	40.0–40.5	3.00	2.82	13.4	–,!	*Secale*
*A. oporinanthum*	11	65 (5.9)	4*x* (100.0%)	4*x*	100.0	32	65	1.458	0.036	1.395–1.504	7.5	7	15	49.5	1.3	47.8–51.4	3.31	3.38	12.4	!,!	*Triticum*
*A. orestis*	1	9 (9.0)	2*x* (100.0%)	2*x*	100.0	–	9	1.928	0.006	–	–	1	1	31.0	–	–	3.71	3.84	15.5	!,!	*Secale*
*A. pallens*	19	121 (6.4)	4*x* (100.0%)	4*x*	100.0	32	121	1.595	0.044	1.527–1.684	9.8	11	11	43.1	1.2	41.6–45.7	3.75	3.75	10.8	!,!	*Vicia*
*A. praescissum*	1	8 (8.0)	2*x* (100.0%)	2*x*	100.0	–	8	1.954	0.071	–	–	1	3	31.6	1.4	–	5.23	4.66	15.8	–,!	*Secale*
*A. pseudostamineum*	1	1 (1.0)	2*x* (100.0%)	2*x*	100.0	16	1	1.404	–	–	–	1	1	22.7	–	–	2.64	2.56	11.4	!,!	*Secale*
*A. rhodopeum*	6	32 (5.3)	2*x* (100.0%)	2*x*	100.0	16	32	1.165	0.035	1.097–1.190	8.1	4	4	31.1	1.2	29.4–31.8	2.93	1.30	15.6	!,!	*Vicia*
*A. rupestre*	6	23 (3.8)	2*x* (*x* = 7, 2, 33.3%), 3*x* (*x* = 7, 1, 16.7%), 4*x* (*x* = 7, 1, 16.7%), 3*x* (*x* = 8, 2, 33.3%)	2*x* (*x* = 7)	34.8	14	8	1.378	0.019	1.365–1.391	1.9	2	6	22.3	0.1	22.1–22.5	3.86	2.72	11.2	!,!	*Secale*
				3*x* (*x* = 7)	17.4	21	4	1.790	0.036	–	–	1	3	29.0	0.6	–	2.86	3.09	9.7	!,!	*Secale*
				4*x* (*x* = 7)	13.0	28	3	2.652	0.002	–	–	1	3	42.9	0.3	–	2.50	2.71	10.8	!,!	*Secale*
				3*x* (*x* = 8)	34.8	24	8	2.379	0.108	2.302–2.456	6.5	2	5	38.6	0.4	–	3.46	3.38	12.9	–,!	*Secale*
*A. telmatum*	1	10 (10.0)	4*x* (10, 100.0%)	4*x*	100.0	32	10	3.131	0.015	–	–	1	7	50.7	0.8	–	3.73	3.18	12.7	!,!	*Secale*
*A. tenuiflorum*	8	40 (5.0)	2*x* (100.0%)	2*x*	100.0	16	40	1.478	0.084	1.396–1.659	17.8	6	9	23.5	0.8	22.5–24.4	3.33	3.68	11.8	!,!	*Secale*
*A. valdesianum*	1	5 (5.0)	2*x* (5, 100.0%)	2*x*	100.0	16	5	1.668	0.019	–	–	1	2	27.3	0.4	–	3.02	3.50	13.7	!,!	*Secale*

Basic descriptive statistics are reported for both RGS and AGS. See M&M section and Table S1 for details. Explanations: Npop = number of populations analysed; Nind (mean) = total number of individuals analysed (and mean per population) on RGS; Ploidy_comp (count, % of Npop) = detected ploidy composition of populations (count, % of total number of populations); Chrom (2n) = somatic chromosome counts (2n) newly counted; Nind (RGS) = number of individuals analysed on RGS; RGS (min–max) = RGS (min–max of population means); Var (%) = interpopulation variation in RGS (%) [(max–min)/avg]; Npop (AGS) = number of populations analysed on AGS; Nind (AGS) = number of indivuduals analysed on AGS; CV (%) stand. = coefficient of variation of the peak of internal standard in AGS; CV (%) sample = coefficient of variation of the peak of *Allium* sample in AGS; Prime AGS = our AGS estimate is the first for the respective ploidy (! = yes, – = no), our AGS estimate is the first by FCM for the respective ploidy (! = yes, – = no); Primary stand. = internal standard used as a primary one for the RGS and AGS estimation; SD = standard deviation. * after a outlier population was excluded

***Allium aetnense* Brullo, Pavone & Salmeri**—The species is a regional endemic of Mt. Etna in Sicily (Brullo et al. 2013). The FCM of six plants from a population on the northern slopes of Mt. Etna revealed a single cytotype identified as diploid (2n = 16, Fig. S1A), consistent with the only previous report (Brullo et al. 2013). Our AGS estimate is the first for the species (Table 1).

***Allium carinatum* subsp. *carinatum****—*The subspecies is the most common member of the informal *A. carinatum* complex (sensu Levan 1933; Stearn 1980). However, in some taxonomic treatments (e.g., Jauzein and Tison 2001, Tison and Foucault 2014) a more complex approach is applied, where *A. flexum* Waldst. & Kit. and *A. consimile* Jordan ex Gren. are treated as independent species. It is native to northern Turkey and most parts of Europe except southwestern and northwestern Europe, Finland, Belarus and Russia (POWO 2024). The FCM of 414 plants from 75 populations revealed the presence of three cytotypes (Table 1): dominant triploids (2n = 3*x* = 24), less frequent diploids (2n = 2*x* = 16) and a very rare cytotype with the RGS corresponding to DNA-tetraploids, detected for the first time in this subspecies. The RGS data in di- and triploids were confirmed by chromosome counts (Fig. S1B,C), whereas this was not possible in the inferred DNA-tetraploids due to poor growth of the plants. Previous studies have reported two ploidy levels (i.e., 2*x*, 3*x*) for this taxon (e.g., Levan 1933; Geitler and Tschermak-Woess 1962; Table S2). Jauzein and Tison (2001) reported a tetraploid count for *A. consimile* from France. Since some authorities (POWO 2024) consider this species to be a synonym of *A. carinatum* (= *A. carinatum* subsp. *carinatum*), this count could support the DNA-4*x* we measured using FCM. However, we agree with Jauzein and Tison (2001) that further study is required to clarify its taxonomic status.

In addition, aneuploids (2n = 25, 26) have rarely been documented in several Austrian populations (Tschermak-Woess 1947; Geitler and Tschermak-Woess 1962). However, later studies (e.g., Speta 1984; Wetschnig 1992), which also detected supernumerary chromosomes in triploid Austrian populations, classified them as B chromosomes. Similarly, Cheshmedzhiev (1973) reported the presence of a B chromosome in a triploid Bulgarian population (Table S2).

**Fig. 1:**
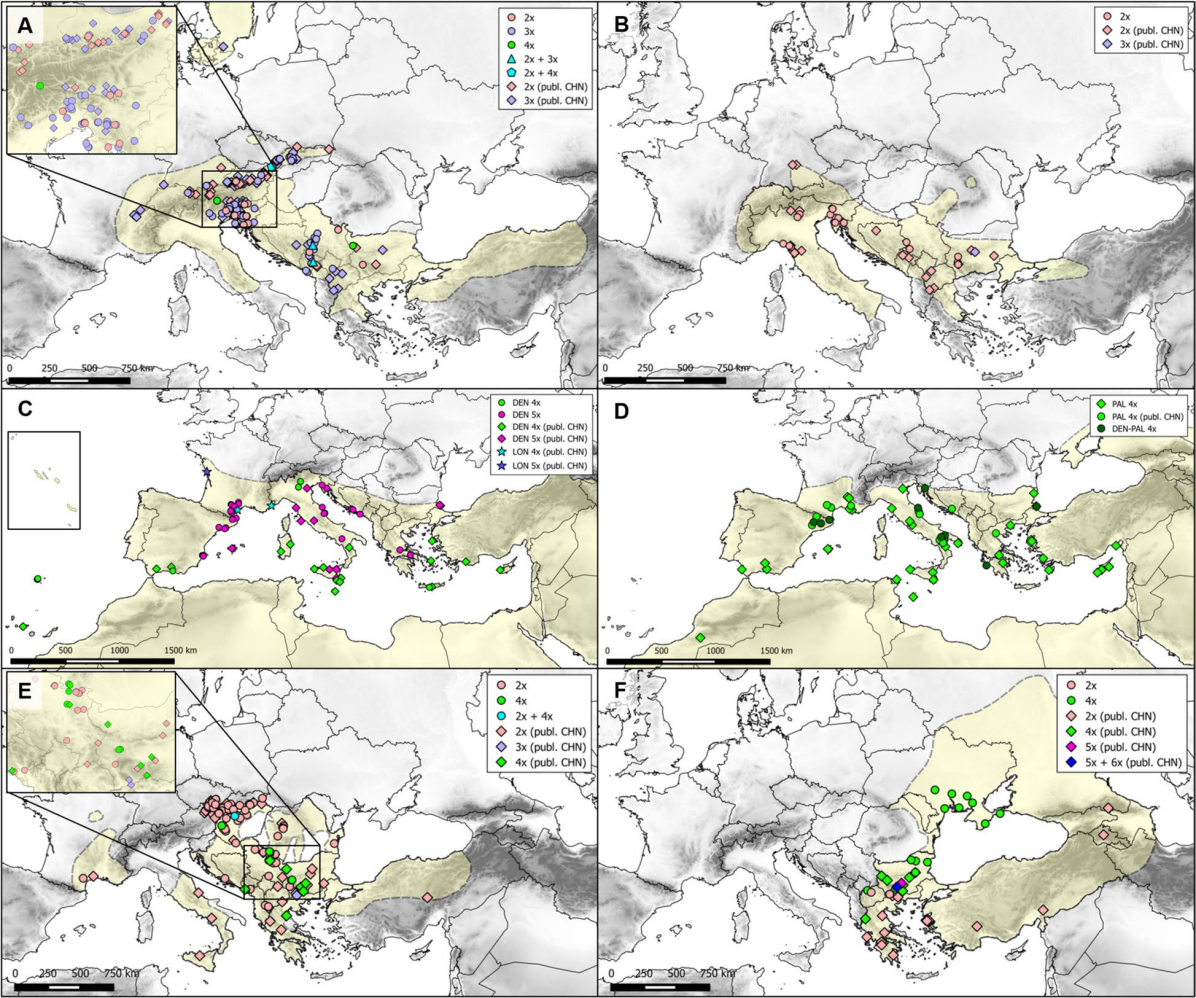
Distribution of ploidy levels of some taxa of *Allium* sect. *Codonoprasum*, based on new and published records [publ. CHN]. **A** *A. carinatum* subsp. *carinatum*, **B** *A. carinatum* subsp. *pulchellum*, **C** *A. dentiferum* [DEN] and *A. longispathum *sensu Jauzein and Tison (2001) [LON], **D** *A. pallens* [PAL] and group of populations assigned to the informal group “*A. dentiferum-pallens*” [DEN-PAL], **E** *A. flavum* subsp. *flavum*, **F** *A. flavum* subsp. *tauricum*. Circles represent new records based on either chromosome number counts or FCM (Table S1), diamonds represent previously published records [publ. CHN]. Mixed-ploidy populations are indicated by ‘+’ between co-occurring ploidies. The approximate range of each taxon (except *A. longispathum* and “*A. dentiferum-pallens*”), based on various sources, is shown in light yellow within the respective map

Pure triploid populations were the most frequent (77.3%) in our data set, followed by pure diploid populations (16.0%). Pure DNA-4*x* and mixed 2*x* + 3*x* and 2*x* +  DNA-4*x* populations were rare, accounting for a total of five populations (Table 1). Previously, only pure cytotype populations had been reported, with triploids being more common than diploids (Table S2). In line with published data, diploids and triploids had a similar geographical distribution, occurring throughout the range of the subspecies (Fig. 1A). Several new national records were found for both di- and triploids (Table S1). Putative DNA-4*x* were found in three countries (Bulgaria, Italy, Slovakia).

**Fig. 2:**
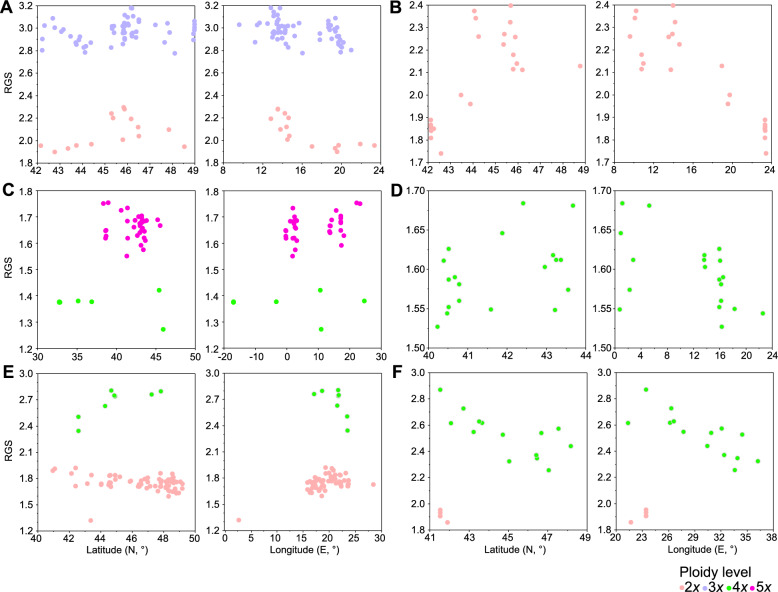
Relationships between relative genome size (RGS) and geography (longitude, latitude) for measured accessions of **A** *A. carinatum* subsp. *carinatum*, **B** *A. carinatum* subsp. *pulchellum*, **C** *A. dentiferum*, **D** *A. pallens*, **E** *A. flavum* subsp. *flavum*, **F** *A. flavum* subsp. *tauricum*. Each ploidy level is shown separately within the plots. Each point represents the mean RGS of the respective population (see Table S1)

The RGS of diploids showed a tendency towards bimodal distribution and a significant geographical pattern of increasing RGS westwards (latitude: r_s_ = 0.423, P = 0.149; longitude: r_s_ = −0.741, P = 0.004, Fig. 2A, Fig. S2). In triploids, RGS increased to the northwest (latitude: r_s_ = 0.345, P = 0.007; longitude: r_s_ = −0.428, P < 0.001; Fig. 2A), but the pattern was more complex, with occurrences of mosaic parapatry or mosaic sympatry of populations with high and low RGS (Fig. S2). The AGS of diploids and triploids were variable, i.e. 31.2–36.9 pg (mean 32.9 ± 1.8 pg) and 45.0–51.7 pg (mean 47.9 ± 1.5 pg), respectively. The AGS of putative DNA-tetraploids was 56.7 pg (Fig. S2). The 1C*x* values decreased with increasing ploidy level (Table 1). Previous estimates of nuclear DNA content based on various techniques for both di- and triploids (Nagl and Fusenig 1979; Labani and Elkington 1987; Ohri et al. 1998; Baranyi and Greilhuber 1999; Šmarda et al. 2019) were within the range of the AGS we measured and followed the spatial patterns we observed (although sometimes reported with incorrect ploidy, as the authors did not count chromosomes), considering the geographical origin of the measured plants (Table S3). Only one of the previously published DNA amounts (2C = 22.4 pg, Bösen and Nagl 1978) was completely different, most probably belonging to another species. Divergent AGS values we measured might suggest the existence of several lineages within this taxon and require additional study employing molecular markers.

***Allium carinatum* subsp. *pulchellum* (Regel) Bonnier & Layens***—*The subspecies is native in southeastern France, southern parts of Central Europe, Italy, and southeastern Europe, extending to western Romania (POWO 2024) and northwestern Turkey (Kollmann 1984; Özhatay 1984). The FCM of 120 plants from 21 populations (Table 1, Table S1) revealed a single cytotype, identified as diploid by multiple chromosome counts (2n = 16; Fig. S1D). This is in line with numerous previous records of diploids from several European countries (Fig. 1B, Table S2), the only exceptions being a triploid count (2n = 24 + 1B; Cheshmedzhiev 1973) and several diploid counts with the presence of a B chromosome (2n = 16 + 1B; Cheshmedzhiev 1973, 1975a) from Bulgaria.

The RGS of diploids showed substantial variation (Table 1), with a significant increase towards the northwest (latitude: r_s_ = 0.620, P = 0.003; longitude: r_s_ = −0.788, P < 0.001; Fig. 2B). The AGS followed the spatial pattern observed in the RGS, ranging from 28.1 to 38.8 pg with a mean of 33.6 ± 3.60 pg (Table 1). The Bulgarian and Serbian populations displayed considerably lower RGS and AGS (−38%) than the other populations (Fig. S2). So far, measurements of the nuclear DNA amount using FEM and FCM have been provided by Labani and Elkington (1987) and Veselý et al. (2012) for samples of unknown origin and southeastern France, respectively (Table S3). Both agree well with the majority of our estimates. As the rather divergent AGS values were confirmed by chromosome counting, there are at least two diploid lineages within the taxon that require further study: one with lower AGS typical of the eastern localities and the other with higher AGS common to the western localities.

***Allium daninianum* Brullo, Pavone & Salmeri**—A widespread representative of the *A. stamineum* species group in the Middle East (Brullo et al. 1996a, 2007). FCM of 10 plants from three Israeli populations (Table S1) revealed a single cytotype, identified as diploid (2n = 16, Fig. S1E). This is consistent with previous records of diploids from Israel and Lebanon (Table S2), with 1–2 B chromosomes reported by Brullo et al. (1996a) from the Coastal Galilee. Our AGS estimate is the first for this species (Table 1).

***Allium dentiferum* Webb & Berthel**.—The species is considered taxonomically critical due to its confusion with *A. longispathum* Redouté. Some authorities (Bartolucci et al. 2024; POWO 2024) and several French authors (Jauzein and Tison 2001; Dobignard and Chatelain 2010; Tison and de Foucault 2014) treat *A. dentiferum* as a synonym of *A. longispathum*, considering some diagnostic characters of the former species (especially the presence of interstaminal teeth) as variable and not distinctive. However, Brullo et al. (1991, 2008) and Brullo and Guarino (2017) consider *A. dentiferum* to be distinct from *A. longispathum*. This taxonomic confusion arises from the fact that both the protologue and the original illustration of *A. longispathum* (Redouté 1811) and the lectotype designated by Wilde-Duyfjes (1976) do not provide clear morphological differentiation from some related species. The treatment on the origin and morphological variation and habitat of the type material of *A. longispathum* by Jauzein and Tison (2001) and the recent photographs of plants assigned to this species from the vicinity of the type localities (Bordeaux and Nantes, France; see Redouté 1811; Wilde-Duyfjes 1976) on iNaturalist (e.g., https://www.inaturalist.org/observations/14161096) suggest that both taxa might be closely related, if not identical. If the two species are indeed the same, the name *A. longispathum* holds priority over *A. dentiferum* (Jauzein and Tison 2001).

During our research (Vojtěchová et al., unpubl. results) we observed two groups of plants: (i) plants that closely resembled the description of *A. dentiferum* (sensu Brullo et al. 2008), (ii) plants that differed from “typical” *A. dentiferum* by shorter (4.0–5.5 mm), apiculate, truncate or subobtuse tepals with a slightly different colour (dirty white with pinkish to purplish strips or strikes), ovary cylindrical-elliptical, smooth or slightly papillose in the upper part, stamens exerted from the perigon and often inconspicuous or absent interstaminal teeths (provisionally labelled as “*A. dentiferum-pallens*”). The second group of plants mix characters of several species, i.e. they partly resemble the description of *A. longispathum* (sensu Jauzein and Tison 2001), but also *A. pallens* L. p. p. (sensu Brullo et al. 2003). Genetic analysis is urgently needed to clarify the relationships within this complex, as the morphological variation observed could be partly due to hybridisation between *A. dentiferum* and *A. pallens*, or introgression of *A. pallens* into *A. dentiferum*, as speculated by Jauzein and Tison (2001).

The FCM of 233 plants from 43 populations of “typical” *A. dentiferum* (Table S1) revealed two cytotypes, confirmed by several chromosome counts (Table 1, Fig. S1F,G): tetraploid (2n = 32) and pentaploid (2n = 40), both also reported in previous reports, which were directly referred to as *A. dentiferum* (Brullo et al. 1991, 2008). However, in contrast to previous reports (Table S2) where tetraploids dominated (69%) over pentaploids (41%), our data suggest the opposite (4*x*: 28.3%, 5*x*: 71.7%, Table 1). We have confirmed previous records of tetraploids from Spain, Italy and Greece (Crete), and added new records from Portugal (Madeira). Brullo et al. (1991, 2008) also recorded tetraploids in Malta, Cyprus and Turkey (Table S2). Pentaploids have been documented in Italy and France (Table S2), with our study adding new records from Spain, Slovenia, Croatia and Greece (Fig. 1C, Table S1). Only pure-cytotype populations were found (Table 1). Several chromosome records attributed to *A. pallens* var. *pallens* in Bulgaria by Cheshmedzhiev (1970, 1975b) may actually belong to *A. dentiferum*, considering the photographs of the analysed plants and the notes on their morphology made by the author (Cheshmedzhiev 1975b). On the other hand, some chromosome reports of Bulgarian plants identified as *A. longispathum* (Cheshmedzhiev 1975c, 1977) probably represent other, presently unidentified species (Table S2). Similarly, Koçyiğit and Özhatay (2011) reported diploid counts for *A. dentiferum* from plants sampled in Turkey, but we consider this count actually pertains to a different species.

Tetraploids showed low and pentaploids moderate variation in the RGS (Table 1). The RGS/AGS of both cytotypes showed a tendency to increase towards the east (RGS; 4*x*: latitude: r_s_ = 0.123, P = 0.772; longitude: r_s_ = 0.344, P = 0.404; 5*x*: latitude: r_s_ = −0.062, P = 0.728; longitude: r_s_ = 0.340, P = 0.053; Fig. 2C, Fig. S2). Our AGS estimates are the first for both ploidy levels, with the 1C*x* values decreasing as ploidy level increases (Table 1).

Twenty-six plants from nine populations assigned to the second group (“*A. dentiferum-pallens*”) were all found to be tetraploid (Table 1, Fig. S1H), consistent with data from France for *A. longispathum* (Jauzein and Tison 2001). In contrast to tetraploid *A. dentiferum*, however, their RGS were more variable (Fig. S2) and mean AGS was slightly lower (Table 1, Table S1). These populations were found in Spain, Italy, Croatia, Bulgaria and Greece (Fig. 1D, Table S1).

***Allium dinaricum* Bogdanović, Anačkov, Ćato, Borovečki-Voska, Salmeri & Brullo**—This recently described species has been observed in several localities in Croatia, Bosnia and Herzegovina, Serbia and Montenegro from rupestrian to mountainous calcareous stands (Bogdanović et al. 2024). The FCM of 13 plants from two populations revealed two slightly different RGS/AGS estimates, probably corresponding to the two cytotypes (Table 1). One cytotype had a 2C value of 35.6 pg, with aneuploid chromosome number (2n = 18, Fig. S1I). The second cytotype had an AGS approximately 3 pg lower (mean 2C = 32.2 pg), a difference that could not be confirmed by chromosome counting due to a lack of available material. However, given the differences observed between the AGS values of the two cytotypes, it can be inferred that the second cytotype represents a diploid cytotype (2n = 16), which was previously reported for this species from the type locality by Bogdanović et al. (2024). Both cytotypes were present in one of the two populations studied, while the second population consisted exclusively of plants belonging to the second cytotype.

***Allium flavum* L**.—The taxonomy and cytogenetics of this species complex are still poorly understood. The most recent taxonomic treatment (POWO 2024) recognizes four infraspecific taxa: subsp. *flavum*, subsp. *ionochlorum* Maire, subsp. *tauricum* (Besser ex Rchb.) K. Richt., and var. *pilosum* (Kollmann & Koyuncu) Koçyiğit & Özhatay. In addition, several other taxa very similar in morphology to members of the *A. flavum* complex have been described from the Eastern Mediterranean and neighbouring regions, most of which are assigned to the informal *A. stamineum* species group (see Brullo et al. 2007). In the Balkan Peninsula, this is the case for *A. guicciardii* Heldr. (see below) and *A. croaticum* Bogdanović, Brullo, Mitić and Salmeri (Brullo et al. 2007; Bogdanović et al. 2008). Many karyological reports of *A. flavum* remain unclear, as they refer to *A. flavum* s.l., without identification at the intraspecific level (Liveri et al. 2019). However, in some cases, knowing the sampling location and the range of the taxon or taxonomic concept used in the respective study, it is possible to infer the infraspecific taxon for which the corresponding chromosome number was published (Table S2).

The nominate subspecies (***A. flavum*** **subsp.** ***flavum***, including var. *minus* Boiss.) is native to southwestern and southern Europe, from southern France to Romania, and Turkey (POWO 2024). The FCM of 337 plants belonging to 76 populations revealed two cytotypes, confirmed by several chromosome counts (Table 1, Fig. S1J,K): diploid (2n = 16, 89.9%) and tetraploid (2n = 32, 10.1%), both also reported in previous reports (Table S2), rarely with the presence of additional 1–3 B chromosomes (see Vujošević et al. 2013 for survey). Cheshmedzhiev (1970) also reported an aneuploid plant (2n = 33) from Bulgaria. In addition, triploids (2n = 24) have rarely been reported from Bulgaria and Greece (Ved Brat 1965; Cheshmedzhiev 1994), but solely for var. *minus* (= *A. webbii* G. C. Clementi). Consistent with previous reports (Table S2), pure diploid populations analysed by us were the most common (89.5%), followed by pure tetraploid populations (9.2%). Diploids were found throughout the range of the species, as previously reported (Fig. 1E). The occurrence of tetraploids has been reported from the Balkan Peninsula (Table S2) and from Granada, Spain (Ruiz Rejón and Sañudo 1976). However, *A. flavum* is not considered to be present in Spain (Aedo 2013) and the chromosome count mentioned likely belongs to another species (Pastor 1985; see also Table S2). The range of tetraploids increased towards the north (Fig. 1E), with new records for Romania, Hungary and Slovakia. We also found a 2*x* + 4*x* mixed population in Slovakia, which is the first report of a mixed-ploidy population for this species (Table 1).

The RGS of diploids showed considerable variation (Table 1), with a significant increase towards the south-east (latitude: r_s_ = —0.329, P = 0.006; longitude: r_s_ = 0.354, P = 0.003; Fig. 2E, Fig. S2). In tetraploids, the RGS showed much less variation (Table 1), but the RGS showed the opposite spatial pattern to the diploids, increasing towards the north-west (latitude: r_s_ = 0.714, P = 0.047; longitude: r_s_ = —0.690, P = 0.058; Fig. 2F, Fig. S2). With the exception of a single measurement, the AGS of diploids followed a unimodal pattern with a mean of 2C = 27.9 pg (Table 1), which confirm previous reports based on both FEM and FCM (Table S3; Baranyi and Greilhuber 1999; Veselý et al. 2012). An unusually low AGS (i.e. 2C = 21.4 pg) was detected in a population sampled near Minerve in southern France, which is geographically isolated from the other analysed populations (Table S1). We interpret this low value as a result of processes acting on isolated and/or marginal populations (Šmarda and Bureš 2010). However, additional samples from southwestern Europe are needed to confirm this pattern.

The AGS of the tetraploids showed a bimodal pattern, with one population group originating from Bulgaria having AGS values between 2C = 38 and 41 pg and the second group (Eastern Central Europe, Serbia) having AGS values between 2C = 42 and 46 pg (Fig. S2). These divergent AGS values were confirmed by chromosome counting (Table S1) and fit well with previous estimates of DNA content using various methods (Labani and Elkington 1987; Ohri et al. 1998; Baranyi and Greilhuber 1999; Ohri and Pistrick 2001), although some of these studies (Labani and Elkington 1987; Ohri et al. 1998; Ohri and Pistrick 2001) erroneously present them as diploid (Table S3). The 1C*x* values decreased with increasing ploidy level (Table 1).

The subspecies ***Allium flavum*** **subsp.** ***tauricum*** **(Besser ex Rchb.) K. Richt.** is native to southeastern Europe, Ukraine, European Russia, Kazakhstan, Caucasian countries, Turkey and Iran (POWO 2024). Plants show rather variable size, shape and colouring of perigon, filaments and anthers (e.g., Vvedensky 1935; Stearn 1980; Ciocârlan 2000; Cheshmedzhiev 2011), which has probably led to the description of several taxa of low taxonomic rank (e.g., Zahariadi 1966; Özhatay and Koçyiğit 2019), but also to species misidentifications (Bogdanović et al. 2011; Tzanoudakis et al. 2019). Recently, Özhatay and Koçyiğit (2019) questioned *A. paczoskianum* as a synonym of *A. flavum* subsp. *tauricum* and consider both taxa as distinct species. Furthermore, the possibility of confusion with morphologically very similar and rarely reported species of the *A. stamineum* group, e.g., *A. guicciardii* Heldr. (Brullo et al. 2007) and *A. croaticum* Bogdanović, Brullo, Mitić & Salmeri (Bogdanović et al. 2008), cannot be excluded in published records. The FCM of 62 plants from 19 populations revealed two cytotypes: karyologically confirmed tetraploids (2n = 32, Fig. S1L) and a cytotype with the RGS approximately 75% of that measured in tetraploids (Table 1). Despite the lack of chromosome counts for these lower RGS plants, we classify them as DNA-diploids, based on a similar pattern of RGS differences between diploids and tetraploids in the closely related *A. flavum* subsp. *flavum*. However, to be sure, additional chromosome counts are desirable.

Four ploidy levels and two aneuploid counts were previously reported for this taxon (Table S2). Tetraploids were the most frequently reported ploidy, followed by diploids. This is consistent with our data, with pure tetraploid populations being the most common (73.7%), followed by pure DNA-diploid populations (26.3%), and no records of cytotype-mixed populations (Table 1). Cheshmedzhiev (1975a, 1982) reported several additional cytotypes, mostly as single records from the Rhodopi Mts and the Thracian lowlands in Bulgaria, i.e., penta- (2n = 40) and hexaploid (2n = 48) as well as tetraploid with 0–3 B chromosomes and aneuploids with 2n = 33 and 34.

Previous records suggested partially different distribution patterns of diploids and tetraploids, with diploids reported from the Caucasus (Pogosian 1983; Magulaev 1992), Turkey (Tanker and Kurucu 1979 sub *A. amphipulchellum* Zahar.; Johnson and Brandham 1997), Northern and Southern Greece (Strid and Franzén 1981; Tzanoudakis and Vosa 1988 sub *A. flavum*) and the island of Lesvos (Karavokyrou and Tzanoudakis 1991 sub *A. flavum*), and tetraploids from Bulgaria (Cheshmedzhiev 1970, 1975a, 1982) and Turkey (Özhatay 1990, 1993). We recorded DNA-diploids as a new cytotype for Bulgaria and North Macedonia, and tetraploids as a new cytotype for Ukraine and North Macedonia (Table S1). Bulgaria represents the most cytotype-diverse region (Fig. 1F, Table S2).

The range of RGS in DNA-diploids was relatively narrow (Table 1), with an almost significant increase towards the east (latitude: r_s_ = −0.154, P = 0.804; longitude: r_s_ = 0.872, P = 0.054; Fig. 2F, Fig. S2). In tetraploids, RGS was variable with a significant increase towards the south-west (latitude: r_s_ = −0.688, P = 0.007; longitude: r_s_ = −0.842, P < 0.001; Fig. 2F, Fig. S2), i.e., opposite to that found in DNA-diploids. The pattern of AGS followed that of RGS. Outlier RGS/AGS values were recorded for two Bulgarian populations, which were approximately 2–4 pg higher than other tetraploid populations (Table S1). This may indicate taxonomic heterogeneity, warranting further investigation. One nuclear DNA estimate using FEM (2C = 39.6 pg; Vakhtina et al. 1977), reported as diploid, is likely tetraploid based on our measurements. The mean 1C*x* of the tetraploids was lower than that of the diploids (Table 1).

***Allium garbarii* Peruzzi***—*The species is endemic to the Calabrian coast in Italy (Peruzzi 2007). The FCM of four plants from the *locus classicus* revealed a single cytotype identified as diploid (2n = 16, Fig. S1M), confirming previous results (Peruzzi 2007). Our AGS estimate is the first for this species (Table 1).

***Allium guicciardii* Heldr**.—This species of the *A. stamineum* group is infrequently mentioned in the literature, despite reports of its occurrence in Central and Northern Greece, as well as Romania (Brullo et al. 2007). It is considered to be a Greek endemic with a limited range by Dimopoulos et al. (2023). We observed a population in northern Greece that clearly belonged to the *A. flavum/A. stamineum* groups, but differed from typical *A. flavum* in having yellow-greenish perigon and stamens white below and violet above. Although the literature provides inconsistent diagnostic characteristics for distinguishing this species from closely related taxa (Brullo et al. 2007; Bogdanović et al. 2008), we provisionally assign this population to *A. guicciardii*. The FCM of eight plants revealed a single cytotype (Table 1), which was not confirmed by chromosome counting (material not available). However, considering the RGS/AGS of these plants and those of the closely related *A. flavum* s. str., we suggest that they are diploid. Previously, diploids (2n = 16; Alden 1976) and tetraploids (2n = 32; Brullo et al. 2007) have been reported for *A. guicciardii*, with both ploidy levels occurring in Greece and tetraploids occurring also in Romania (Brullo et al. 2007). Our AGS estimate is the first for this species (Table 1).

***Allium hermoneum* (Kollmann & Shmida) Brullo, Guglielmo, Pavone & Salmeri**—The species is reported to occur in the alpine belt of the Anti-Lebanon Mts in Syria, Israel and Lebanon (Kollmann and Shmida 1977; Brullo et al. 2007; Danin and Fragman-Sapir 2016). FCM analysis and chromosome counting of two plants from two micropopulations between the peaks of Mt. Habushic and Mt. Hermon (Israel) revealed a single cytotype, identified as tetraploid (2n = 32, Fig. S1N). Previously, Shmida and Kollmann (1977) and Kollmann (1985) reported both diploid and tetraploid chromosome numbers from samples collected at the type locality (Mt. Hermon). Our AGS estimate is the first for this species (Table 1).

***Allium karsianum* Fomin**—The range of this species is restricted to northeastern Turkey and the Transcaucasus (POWO 2024). It is closely related to *A. kunthianum* Vved., and distinguishing between the two can be challenging due to inconsistencies in diagnostic characters in regional floras, and the frequently reported intermediate forms (e.g., Vvedensky 1935; Oganesian and Agababian 2001). The FCM of five plants from three populations from Georgia and Armenia revealed a single cytotype (Table 1), identified as diploid (2n = 16, Fig. S1O), confirming previous diploid records from Armenia (Pogosian 1983) and Turkey (Özhatay 1993). Our AGS estimate for the three populations of diploids was very narrow, around 2C = 35.4 pg (Table 1). There is only one previous AGS report by FEM (2C = 28.5 pg) for diploids of unknown origin by Vakhtina et al. (1977). However, this value differs from our data.

***Allium kunthianum* Vved.***—*This species is closely related to *A. karsianum* (see above), and has been reported to occur in Iran, North Caucasus, Transcaucasus and Turkey (POWO 2024). The FCM of ten plants from three populations from Georgia revealed a single cytotype, identified as diploid (2n = 16, Fig. S1P), confirming previous diploid records from Georgia (Gagnidze et al. 2015) and Armenia (Pogosian 1983; Table S2). Vakhtina and Kudryashova (1985) also reported a tetraploid count from Armenia. The report of 2n = 14 by Vakhtina (1964) is not considered here (see Table S2 and notes on *A. rupestre* below). Our AGS estimate for three populations of diploids was very narrow, around 2C = 32.4 pg (Table 1). There is only one previous AGS report by FEM (2C = 35.1 pg) for diploids of unknown origin by Vakhtina et al. (1977), which is rather closer to our AGS of *A. karsianum*.

***Allium melanantherum* Pančić***—*This rare Balkan endemic species occurs in Bulgaria, Serbia, Kosovo, North Macedonia and northern Greece (Andersson 1991; Anačkov 2009; Assyov et al. 2012; Teofilovski 2021; Dimopoulos et al. 2023; POWO 2024). The FCM of 14 plants from two nearby populations in the Rila Mts (Bulgaria) revealed a single cytotype, identified as triploid (2n = 24, Fig. S1Q). Our chromosome count confirms previous triploid records (some of which indicate the presence of 0–1 B chromosomes) from Bulgaria, from which diploids (2n = 16) and tetraploids (2n = 32) have also been reported (Cheshmedzhiev 1970, 1971, 1976, 1979, 1992). Triploids have also been reported from northern Greece (Tzanoudakis and Vosa 1988, Table S2). Our AGS estimate is the first for triploids (Table 1). Previously, AGS of tetraploids (2C = 47.5 pg) was estimated using FEM (Ohri et al. 1998; Ohri and Pistrick 2001). The 1C*x* value of tetraploids were found to be lower than that of triploids (Fig. 4, Table S3).

***Allium oporinanthum* Brullo, Pavone & Salmeri***—*The species is considered a northwestern Mediterranean species, occurring in Spain, France (Brullo et al. 1997a) and the Aosta Valley in northwestern Italy (Rey et al. 2015). The FCM of 65 plants from 11 populations revealed a single cytotype, which was identified as tetraploid (2n = 32, Table 1, Fig. S1R), confirming previous tetraploid records from several localities in Spain and France (Brullo et al. 1997a; Jauzein and Tison 2001). The range of RGS and AGS values were relatively narrow, and our AGS estimate is the first for this species (Table 1).

***Allium orestis* Kalpoutz., Trigas & Constantin.***—*The species was described from the Parnon Mt. and Taigetos Mt. of the southern Peloponnese, Greece (Kalpoutzakis et al. 2012). The FCM of nine plants from the *locus classicus* resulted in a single cytotype, which was identified as diploid (2n = 16) by the authors of the species description (Kalpoutzakis et al. 2012). Our AGS estimate is the first for this species (Table 1).

***Allium pallens* L.**—Widespread semi-ruderal species in the Mediterranean (Brullo et al. 2003). FCM of 121 plants from 19 populations, clearly matching the species description in Brullo et al. (2003), yielded a single cytotype identified as tetraploid (2n = 32, Table 1, Fig. S1S). Our chromosome counts confirm previous tetraploid records from several European, Turkish and north-African localities (e.g. Jauzein and Tison 2001; Brullo et al. 2003; Fig. 1D), frequently referred under the names *A. coppoleri* Tineo or *A. stearnii* Pastor & Valdés (Table S2). Reported diploid records (2n = 16, Table S2), e.g. from Spain (e.g., Pastor 1982; Ruíz Rejón et al. 1980, 1986), Greece (Karavokyrou and Tzanoudakis 1991) and Turkey (Ved Brat 1965) likely belong to other, presently unidentifiable, species and require further study, owing to frequent misidentification or misinterpretation of the species (Brullo et al. 2003), and are not presented in the map (Fig. 1D). The range of RGS and AGS values of tetraploids was relatively wide, with a clear increase towards the north-west (RGS: latitude: r_s_ = 0.422, P = 0.071; longitude: r_s_ = −0.547, P = 0.015; Fig. 2D, Fig. S2). The AGS estimate is the first for this species (Table 1).

***Allium praescissum* Rchb***.—*The species is distributed from east of the Dnieper River in Ukraine to western Siberia and typically occurs on saline soils (Dobrochaeva et al. 1999; Seregin 2007; Sinitsina 2019). The FCM of eight plants from a Russian population revealed a single cytotype. Despite the lack of chromosome counts for these plants, we classify them as DNA-diploids, based on the similarity of our AGS estimates (2C = 31.6 pg, Table 1) to the karyologically verified nuclear DNA content using FEM (2n = 16, 2C = 28.5 pg) by Vakhtina et al. (1977). Zakirova and Nafanailova (1988) reported diploids also from Kazakhstan (Table S2).

***Allium pseudostamineum* Kollmann & Shmida***—*This endemic species is native to Israel, Syria and Lebanon (Brullo et al. 2007; POWO 2024) and was described from Mt. Hermon in the Anti-Lebanon Mts (Kollmann and Shmida 1977). FCM analysis of a single plant from the population between the peaks of Mt. Habushic and Mt. Hermon (Israel) revealed a single cytotype, identified as a DNA-diploid, consistent with the previous report of 2n = 16 from the type locality (Shmida and Kollmann 1977). Our AGS estimate is the first for this species (Table 1).

***Allium rhodopeum* Velen.***—*This species is native to Bulgaria, Serbia, North Macedonia, Albania, Greece and Turkey (Brullo et al. 1998; Barina and Pifkó 2011; Nikolov 2021; POWO 2024). The FCM of 32 plants from six populations sampled in Bulgaria, North Macedonia and Greece revealed a single cytotype identified as diploid (2n = 16, Table 1, Fig. S1T). This confirms previous diploid records from other regions of Bulgaria (Cheshmedzhiev 1970, 1973), Greece (Brullo et al. 1998) and Turkey (Özhatay 1990, 1993). Ricci (1965) published tetraploid count (2n = 32) in a plant of unknown origin but we omit this count due to uncertainty of the species determination. Our AGS estimate is the first for this species (Table 1).

***Allium rupestre* Steven***—*The species occurs in Crimea, Turkey and Caucasus (Vvedensky 1935; POWO 2024)*.* The FCM of 23 plants from six populations sampled in Georgia and Crimea revealed four distinct RGS/AGS groups. Most of our AGS estimates are the first for the species (Table 1). Three groups with increasing AGS mean of 2C = 22.3, 29.0 and 42.9 pg probably represent a polyploid series with basic chromosome number *x* = 7 and chromosome numbers 2n = 2*x* = 14, 2n = 3*x* = 21 and 2n = 4*x* = 28, respectively (Fig. S1U, V, W). The 2n = 14 is most likely the first such count for the species. However, Vakhtina and Kudryashova (1985) commented on the possibility that a count of 2n = 14 for *A. kunthianum* by Vakhtina (1964) might actually belong to *A. rupestre*. Chromosome counts of 2n = 21 and 2n = 28 have already been published for individuals sampled in the Crimea (Ukraine) and the foothills of the Caucasus (Georgia), respectively (Vakhtina and Kudryashova 1985; Table S2).

Another group with an AGS of 2C = 38.6 pg, intermediate between the AGS of the tri- and tetraploids (see above) is represented by plants from a population originating from Georgia (Table 1), for which we counted 2n = 24 (Fig. S1X). A very similar nuclear DNA amount (2C = 37.8 pg) was also measured for plants originating from the other Georgian locality (Borjomi), from which a chromosome number 2n = 24 was previously reported (Ohri et al. 1998; Ohri and Pistrick 2001; Table S3). The authors consider this chromosome count to be triploid, based on *x* = 8 and two diploid reports (2n = 16) from Armenia (Pogosian 1990) and Turkey (Özhatay 1993). These rare records suggest a rather complicated evolution within the species and require more detailed research over the whole Caucasian region.

***Allium telmatum* Bogdanović, Brullo, Giusso & Salmeri**—The species is endemic to the northwestern Croatian coast (Bogdanović et al. 2009). The FCM of ten plants from one population revealed a single cytotype identified as tetraploid (2n = 32, Fig. S1Y). Our chromosome count confirms previous records from two localities (Bogdanović et al. 2009) close to newly sampled locality. Our AGS estimate is the first for this species (Table 1).

***Allium tenuiflorum* Ten.***—*This Mediterranean species occurs in southern Europe from southeastern France to Bulgaria (Brullo et al. 2003). However, the species is critical because of its morphological similarity to some other species such as *A. pallens* and *A. dentiferum* (Jauzein and Tison 2001; Brullo et al. 2003). The taxonomic status of the eastern Balkan populations (e.g., Bulgaria) also requires further study. FCM of 24 plants from five populations sampled at the southern edge of its range in Italy (cf. Brullo et al. 2003) and 16 plants from three localities in Croatia (Istria, N Dalmatia) yielded a single cytotype identified as diploid (2n = 16, Table 1, Fig. S1Z), confirming previous diploid records (rarely with 1–6 B chromosomes) from Italy (e.g., Marcucci and Tornadore 1994; Jauzein and Tison 2001; Brullo et al. 2003; Peruzzi 2003; Tornadore and Marcucci 2005), Croatia (Puizina et al. 1997) and Bulgaria (Cheshmedzhiev 1975c). In addition, Fernandes and Queiros (1971) also reported a diploid count, though from a plant of unknown origin, so this count should be considered doubtful. Apart from diploids, there are two records of triploids, one from Hyères in southern France (Jauzein and Tison 2001) and one from Apulia in southern Italy (Tornadore 1981). The pentaploids (2n = 40) reported by Vosa (1976) from central Italy clearly belong to another species, most probably *A. dentiferum*. The ranges of RGS and AGS of the diploids we analysed were wide, with a weak tendency of Croatian populations towards slightly higher GS compared to most Italian populations (Table S1). Our AGS estimate is the first for this species (Table 1).

***Allium valdesianum* Brullo, Pavone & Salmeri***—*The species is endemic to the alpine belt of Sierra Nevada Mts in Spain (Brullo et al. 1996b). The FCM of five plants from a population in the northwestern part of the Sierra Nevada Mts revealed a single cytotype identified as diploid (2n = 16, Fig. S1AZ), in agreement with the only previous diploid record from the *locus classicus* (Brullo et al. 1996b), which is almost identical to our locality. Our AGS estimate is the first for this species (Table 1).

### Ploidy variation and frequency in *Allium* sect. *Codonoprasum*: overview

**Fig. 3:**
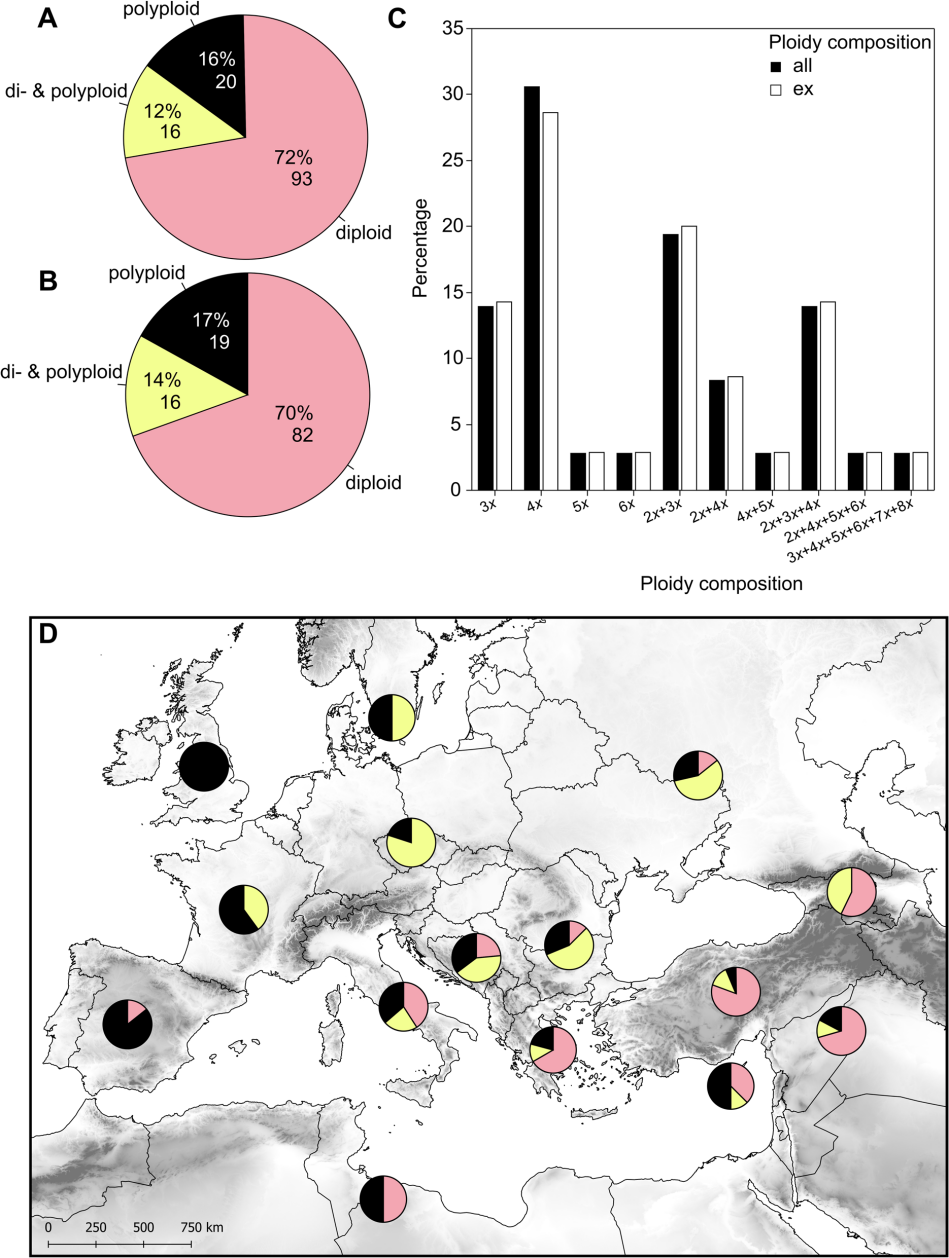
Ploidy level composition of taxa of *Allium* sect. *Codonoprasum.* Frequency (both absolute and relative) of ploidy categories (di-; di- & polyploid; polyploid) with **A** all revised taxa with at least one chromosome count and **B** with 13 taxa excluded based on Özhatay and Koçyiğit (2019). **C** Ploidy level composition of the respective di-&polyploid and polyploid taxa, both with (all, *n* = 37 taxa) and without accounting for some taxa excluded (ex, *n* = 36 taxa) based on Özhatay and Koçyiğit (2019). **D** Frequency distribution of diploid, di- & polyploid, and exclusively polyploid taxa (for colours see (**A**)) in the respective regions (from east to west: Caucasus without RUS; Near East without TUR; TUR; CYP; UKR + RUS + BLR; BGR + ROU; GRC incl. Aegean islands; Former YUG; ITA; Central Europe; Northern Europe; North Africa; FRA; GBR + IRL; ESP + PRT). For details see Tables S1, S2, S4, S5

The issue of high chromosomal variation in the genus *Allium* has been a topic of interest since the benchmarking studies of Levan (1931, 1933). A recent review found that only 3.2% of *Allium* species are pure polyploids (exclusively 4*x*), but about 30.2% exhibit intraspecific variation in ploidy levels, up to eight ploidies (2*x*−10*x*; Han et al. 2020). However, only a subset of species of *A*. sect. *Codonoprasum,* sometimes even with inaccurate cytotype composition, was included in Han’s review. In our study, almost half (44%) of the taxa we screened using FCM were polyploid or di- & polyploid, with tetraploids/DNA-tetraploids being the most common polyploid cytotype (Table 1). The synthesis of our new data (Table S1) and the published chromosomal/DNA-ploidy data for 166 recognised taxa (species, subspecies) of *A.* sect. *Codonoprasum* (Tables S2, S4, all taxa summarised in Table S5) showed that 37 taxa (22.3% of total) lack ploidy data. Moreover, many older chromosome records, especially those referring to members of the *A. paniculatum* complex (e.g., *A. paniculatum* L., *A. fuscum* Waldst. & Kit.), should be considered with caution or even excluded due to species misconception/misidentification (Salmeri et al. 2016; Vojtěchová et al. 2023; see Tables S2, S4 for details). Considering only reliable chromosome data for taxa with at least one chromosome record (129 taxa), diploid taxa are dominant (72.1%), while di- & polyploid and exclusively polyploid taxa are less common, occurring at similar frequencies of 12.4 and 15.5%, respectively (Fig. 3A). After excluding taxa recently transferred to another section according to Özhatay and Koçyiğit (2019), 82 out of 117 taxa with at least one chromosome count are diploid (70.1%), 16 di- & polyploid (13.7%) and 19 are exclusively polyploid (16.2%, Fig. 3B). While the proportion of diploid species is consistent with the overall pattern observed in the entire genus *Allium* (Han et al. 2020), the proportions of di- & polyploid, and exclusively polyploid taxa in *A*. sect. *Codonoprasum* differ from those found by Han et al. (2020). We attribute these differences to a narrower and more critical species concept, as well as a more detailed survey in our study compared to Han et al. (2020).

A detailed analysis of ploidy composition in di- & polyploid and exclusively polyploid taxa in *A*. sect. *Codonoprasum* shows that 4*x,* 3*x*, 2*x* + 3*x* and 2*x* + 3*x* + 4*x* taxa are dominant, while other ploidy levels or combinations are rare (Fig. 3C, Table S5), e.g. pentaploid *A. pseudotelmatum* Duchoslav & Jandová, hexaploid *A. exaltatum* (Meikle) Brullo, Pavone, Salmeri & Venora, and up to hexaploid *A. flavum* subsp. *tauricum*. The most intriguing case is *Allium oleraceum* L., which shows a wide ploidy range from tri- to octoploids (for details see Duchoslav et al. 2020). These results contrast with Han et al. (2020), who identified 4*x* and 2*x* + 4*x* combination as the most common (48.1 and 45.9%, respectively) among di- & polyploid, and exclusively polyploid *Allium* species. In addition, our synthesis reveals some new cytotype combinations not covered by Han et al. (2020), suggesting a higher cytotype diversity within *A*. sect. *Codonoprasum*. This also suggests that the true proportion of mixed-ploidy species may be underestimated due to the sample-size limitations of classical chromosome counting (Duchoslav et al. 2020), as mixed populations may not be detected at all, although reports of different cytotypes coexisting in the same regions in several taxa (Tables S1, S2, S4) might also suggest this. In this respect, the use of more convenient and efficient methods like FCM is beneficial (Siljak-Yakovlev et al. 2020; Sliwinska et al. 2022), although its application in *Allium* is still scarce (but see Duchoslav et al. 2010, 2013, 2020; Vojtěchová et al. 2023, 2024).

Contrary to our expectations, we recorded only four mixed-ploidy populations, representing 1.3% of the populations newly analysed here, and these were found in only two taxa: *A. carinatum* subsp. *carinatum* and *A. flavum* subsp. *flavum* (Table 1). When we include previously published population-level ploidy data (Table S4), only three other taxa in the section (*A. oleraceum*, *A. paniculatum*, *A. marginatum* Janka) have documented mixed-ploidy populations. Apart from the unique case of *A. oleraceum* with complex population ploidy structure (Duchoslav et al. 2010, 2020), 2*x* + 3*x* and 2*x* + 4*x* populations were only rarely found in the other four taxa (Table S4). This is noticeably lower than the 16.1% of populations with multiple cytotypes found in 39 mixed-ploidy species (Kolář et al. 2017). However, in contrast to our study, the majority of studies reviewed by Kolář et al. (2017) were specifically designed to detect within-population ploidy diversity by sampling a high number of individuals per population (16 *vs.* 5 on average, Kolář et al. 2017*vs.* our study) and extensively covering the entire area of the population. This sampling strategy has been shown to increase the probability of detecting multiple ploidies (Duchoslav et al. 2020). Therefore, the combination of intensive within-population sampling and FCM may increase the frequency of mixed-ploidy populations, especially in taxa with enhanced vegetative reproduction (e.g., through bulbils or bulblets), which may help different cytotypes avoid the process of minority cytotype exclusion in local sympatry (Herben et al. 2017; Kolář et al. 2017).

The spatial pattern of richness of diploid, di- & polyploid and exclusively polyploid taxa from *A*. sect. *Codonoprasum* across Europe and adjacent regions is shown in Fig. 3D. Diploid taxa dominate in the eastern Mediterranean, where they represent more than 75% of all taxa with documented chromosome counts. The proportion of diploid taxa gradually decreases both westwards and northwards, with only exclusively polyploid taxa and di- & polyploid taxa present in northern and northwestern Europe. The dominance and diversity of diploid taxa in the Eastern Mediterranean and the adjacent Irano-Turanian region could be attributed to these regions being considered evolutionary centres for the genus/section (Hanelt 1996; Fritsch and Friesen 2002; Friesen et al. 2006). It could be speculated that the ancestors of extant species migrated westward from their ancestral area in the past, e.g. across newly opened dry environments during the Messinian salinity crisis (Krijgsman et al. 1999; Trájer et al. 2021). These migrations were followed by evolution in isolation in newly colonised areas after the marine transgression at the end of the Tertiary (Garcia-Castellanos et al. 2009). This east–west phylogeographical divide has frequently been inferred, and sometimes dated in various plant groups (Nieto Feliner 2014). Several authors (e.g., Bogdanović et al. 2009; Salmeri et al. 2016) have suggested that this scenario is particularly likely in autumn-flowering taxa of *A*. sect. *Codonoprasum*, where all but one (*A. apolloniensis* Biel et al.) extant taxa in Eastern Mediterranean are diploid, while those occurring westward are (paleo-)polyploids (Brullo et al. 1997b; Bogdanović et al. 2009; Özhatay et al. 2018). These groups form distinct subclades in the concatenated nr-ITS and cp-DNA tree (Salmeri et al. 2016).

A high proportion of diploid taxa, often endemic, is also associated with regions of climatic, geological and topographical complexity that experienced less severe glaciation effects during the Quaternary (Hughes and Woodward 2017; Noroozi et al. 2019), e.g., Turkey, Greece, southern Italy, and Sicily (Peruzzi et al. 2015). This scenario is considered to be the most parsimonious explanation for the high species diversity and endemism in many species complexes across the Mediterranean region (Nieto Feliner 2014).

In contrast, the northern regions of Europe are dominated by higher ploidies of di- & polyploid taxa (e.g., Fig. 1A) or exclusively polyploid taxa (Fig. 3D). The most widespread taxa of the section are also polyploids (e.g., *A. oleraceum*, Duchoslav et al. 2020; *A. dentiferum*, *A. pallens*; see Fig. 1C, D). The observed increase of polyploid taxa/cytotypes towards north fits well with global trends (Rice et al. 2019) and reflect the strong influence of the Quaternary glacial cycles on the flora of central and northern Europe (Hewitt 1999). This also highlights the adaptive advantage of polyploidisation and hybridisation within the section (Duchoslav et al. 2020), which might have resulted from secondary contacts during postglacial range expansions from different refugia (Schmitt 2007). The high proportion of polyploid and mixed-ploidy taxa within the section is considered indicative of ongoing diversification (Tzanoudakis and Vosa 1988; Hanelt 1996). This diversification likely contributed to the ecological radiation of *A*. sect. *Codonoprasum* into newly emerging habitats, especially those with mesic climates and/or fertile conditions, as demonstrated by Han et al. (2020) for the genus *Allium*. Indeed, many widely distributed polyploid taxa of this section dominate in mesic and/or fertile, frequently anthropogenic, disturbed habitats (Brullo et al. 2008; Duchoslav et al. 2020; Vojtěchová et al. 2024). Also, relatively high frequency of odd ploidy taxa and odd cytotypes within taxa (especially 3*x*) observed within the section is unusual in polyploid plants (Kolář et al. 2017), as odd ploidy causes meiotic irregularities leading to a reduction of the seed set and thus lowering the fitness of newly emerging polyploids (Ramsey and Schemske 1998, 2002). However, polyploid members of the section either form or increase their vegetative reproduction via production of aerial bulbils or underground bulblets (e.g., triploid *A. corsicum*, Jauzein et al. 2002; polyploid *A. oleraceum*, Fialová and Duchoslav 2014), which allow them to establish, persist in their habitats, and disperse to new areas (Hörandl 2009), overcoming reproductive constraints associated with odd ploidy.

Robust data explaining the origin of polyploid cytotypes within di- & polyploid and exclusively polyploid species within *A.* sect. *Codonoprasum* are limited and mostly based on interpretation of mitotic chromosome arrangements (e.g., Pastor 1982; Bogdanović et al. 2011) or GS (e.g., Vojtěchová et al. 2023, 2024). Therefore, the origin of most taxa remains unclear, with only general hypotheses presented in literature. Both evolutionary pathways of polyploid formation (auto- and allopolyploidy) are briefly discussed. For example, some tetraploid species are considered to be either autotetraploid (e.g., *A. occultum* Tzanoud. & Trigas; Tzanoudakis and Trigas 2015) or allotetraploid (e.g., *A. apergii* Trigas, Iatroú & Tzanoud.; Trigas et al. 2010). For some di- & polyploid species containing tri- and/or tetraploids in addition to diploids (e.g., *A. paniculatum*, *A. marginatum*), an autopolyploid origin is postulated based on both GS and molecular markers (Vojtěchová et al. 2023), whereas for polyploid 4*x*−5*x* *A. dentiferum* and 3*x*−8*x* *A. oleraceum*, an allopolyploid origin is most likely (Brullo et al. 2008; Duchoslav et al. 2020 and references therein).

### Rare dysploidy in *A. rupestre*

Traditionally, several basic chromosome numbers have been distinguished within the genus *Allium*, ranging from *x* = 7 to *x* = 11 (Jones and Rees 1968; Friesen et al. 2006). The most common is *x* = 8, which is dominant in most subgenera (Fritsch et al. 2010), including the subgenus *Allium* (Ved Brat 1965; Peruzzi et al. 2017; Han et al. 2020), and has also been inferred as the ancestral basic chromosome number by ancestral state reconstruction (Peruzzi et al. 2017). Consistent with these findings, all but one of the species we analysed had *x* = 8. In our study of *A. rupestre* populations, we observed a chromosome number of 2n = 24, corresponding to 3*x* with *x* = 8, together with a polyploid series (2*x–*3*x*–4*x*) with 2n = 14, 21 and 28 (see also Vakhtina and Kudryashova 1985). These results indicate a descending dysploidy leading to *x* = 7, which is very unusual for members of *A*. sect. *Codonoprasum*, as well as for the subgenus *Allium* (Mathew 1996; Peruzzi et al. 2017; Babin and Bell 2022), where only a few dysploid (*x* = 7) species are known, e.g., from the *A.* sect. *Cupanioscordum* Cheshm. (Brullo et al. 2015; Trigas et al. 2017), which is sister to the *A*. sect. *Codonoprasum* (Li et al. 2010).

### General patterns of GS variation within the *Allium* sect. *Codonoprasum*

**Fig. 4:**
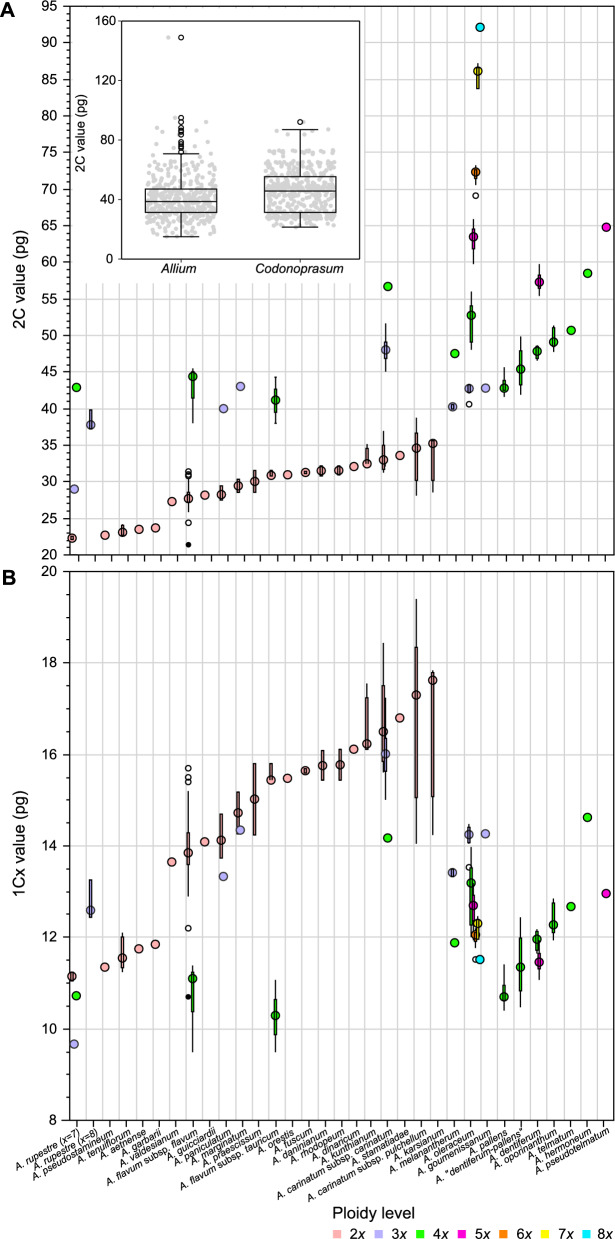
Genome size of taxa in the *Allium* sect. *Codonoprasum*. All available data on GS (population means) meeting the strict criteria (see M&M) were used for the analyses. **A** Absolute genome size (AGS, 2C values, pg). In the inset, variation in AGS (2C values) within the whole genus *Allium* (data from Leitch et al. 2019) and *A*. sect. *Codonoprasum* (Tables S1, S3) are compared. **B** Monoploid genome size (1C*x* values, pg). Taxa are ordered along the x-axis from left to the right according to an increasing median of 2C values of the lowermost ploidy. Where multiple population measurements are available for a given taxon, a boxplot of GS is presented, separately for each ploidy level. The median AGS is represented by a coloured circle. In the case of only one measurement per taxon/ploidy, only the coloured circle representing the measured value is shown

Our research represents the first comprehensive study of GS in *A.* sect. *Codonoprasum*, increasing the number of taxa with known GS threefold compared to the latest version of the Plant C-value Database (Leitch et al. 2019). New AGS data revealed a 2.6-fold difference in nuclear DNA content, ranging from 2C = 22.3 pg, which is the lowest known AGS within this section, to 2C = 58.5 pg (Table 1, Fig. 4A). Previously, Jones and Rees (1968) reported an even lower 2C value (18.4 pg) than the lowest we measured within this section, but this record certainly belongs to other species, probably from another section (Vojtěchová et al. 2023). When considering previous AGS records from other species in this section (Leitch et al. 2019), including the highly polyploid *A. oleraceum* (Duchoslav et al. 2013), the difference in GS increases to 4.1-fold, with the maximum known 2C = 92.1 pg in octoploid *A. oleraceum* (Fig. 4A, Table S3). This variation covers most of the known range of AGS in the whole genus (Fig. 4A inset), with the exception of several species from other sections that either have lower AGS, with the lowest AGS (2C = 15.2–16.9 pg) found in *A. schoenoprasum* L. (Jones and Rees 1968; Baranyi and Greilhuber 1999), or an extremely high AGS record in *A. validum* S. Watson (2C = 148.9 pg, Ohri et al. 1996). Based on these findings, members of *A.* sect. *Codonoprasum* belong to plant groups with large or very large GS sensu Leitch et al. (1998).

The observed GS variation within *A.* sect. *Codonoprasum* is mainly driven by polyploidy (Fig. 4A), which plays a significant role in the evolution of the genus (Friesen 1992; Hanelt et al. 1992; Gurushidze et al. 2012; Han et al. 2020; Wang et al. 2023), and is also crucial for its ecological radiation (Wu et al. 2010; Han et al. 2020; Wang et al. 2023). Increased chromosome number due to polyploidy resulted in higher 2C values but lower 1C*x* values in all but two cases of single ploidy in taxa examined with multiple ploidy levels (Fig. 4A, B). This pattern of GS reduction in polyploids, often associated with DNA loss, is well-documented and may support the success of autopolyploid and allopolyploid speciation (e.g., Ozkan et al. 2003; Leitch and Bennett 2004; Poggio et al. 2014; Wang et al. 2021). Differences in 1C*x* values between diploids and tetraploids, such as those observed in *A. flavum* and *A. carinatum*, may reflect the age of the polyploids. A small reduction in 1C*x* is typical of younger polyploids (neopolyploids), where genome downsizing processes have not yet occurred (Ekrt et al. 2010; Bressler et al. 2017; Wang et al. 2021; Pungaršek and Frajman 2024). This may also explain why the 2C values of some (neoauto-)triploids, e.g., in *A. paniculatum* and *A. marginatum* (see Vojtěchová et al. 2023 for discussion), are similar to those of tetraploids of other species, despite the similarity of 2C values among the respective diploids (Fig. 4B). Alternatively, some tetraploid cytotypes may represent allopolyploids formed through hybridization between diploid species with different 2C values. In *A. rupestre* (*x* = 7), tetraploids had a higher 1C*x* than triploids, but this may be related to the effect of dysploidy observed in this species (see above).

GS variation in *Allium* may also be affected by phylogenetic signals (Wang et al. 2023), suggesting that GS evolution may reflect phylogenetic relationships (e.g., Weiss-Schneeweiss et al. 2006; Chrtek et al. 2009; Hutang et al. 2023). Unfortunately, only a limited number of *A*. sect. *Codonoprasum* members have been sequenced, and the scarcity of GS estimates currently precludes a full reconstruction of GS evolution along the phylogeny. Two main clades [A, B] have been identified in the limited number of accessions of *A*. sect. *Codonoprasum* analysed so far, based on ITS and/or the combined ITS and trnH-psbA data (fig. 6 in Salmeri et al. 2016; fig. 2 in Vojtěchová et al. 2024). Interestingly, when mapping the available 2C values of diploid members of both clades onto these phylogenetic trees, diploid species with larger genomes (2C > 27 pg, Fig. 4A) and smaller genomes (with 2C < 25 pg) are recorded within the A and B clades (sensu Salmeri et al. 2016), respectively. When considering monoploid GS, sequenced taxa with 1C*x* > 12 pg (e.g., *A. flavum*, *A. paniculatum*) are placed in the clade A, whereas those with 1C*x* < 12 pg (e.g., *A. dentiferum*, *A. pallens*, *A. tenuiflorum*) are included in the clade B. Any further consideration of the ancestral state and possible direction of GS evolution in the two main clades will require additional sequencing, as current phylogenetic trees are constrained by incomplete taxon sampling.

### Intraspecific intra-ploidy variation in GS and its ecological and taxonomic consequences

Traditionally considered to be a stable trait at the plant species level (Ohri 1998), GS can actually exhibit significant intraspecific variability among individuals and populations (Šmarda and Bureš 2010). Published chromosome/karyotype reports suggest that the higher intra-ploidy GS variation found in some taxa (e.g., *A. flavum*, Vujošević et al. 2013) could be partly explained by chromosomal changes, such as aneuploidy, or the presence of accessory B chromosomes. However, supernumerary chromosomes tend to have only a minor impact on individual GS, particularly in plants with higher GS (Levin et al. 2005; Chumová et al. 2016; but see Leitch et al. 2009), which is common in *Allium* species.

The primary driver of genome expansion within a given ploidy level is the proliferation of transposable elements (Feschotte and Pritham 2007; Lisch 2013). Conversely, DNA loss is often associated with unequal homologous recombination or illegitimate recombination events (Bennetzen et al. 2005). Such variation may indicate the response of GS to environmental constraints across ecogeographic gradients (Knight et al. 2005) or during range expansion and invasion (Guo et al. 2024), often accompanied by genetic drift (Cang et al. 2024). Alternatively, intraspecific GS variation could reflect a complex evolutionary history (Loureiro et al. 2010), potentially involving unrecognised phylogenetic components (Greilhuber 1998, 2005).

In our study, we recorded considerable intraspecific intra-ploidy GS variation in several taxa (e.g., *A. carinatum*, *A. flavum*, Table 1, Figs. 2, 4A, B), with samples collected from substantial parts of their geographical range. Longitude, which can be considered a surrogate for the continental climate gradient in Europe (Mikolaskova 2009) and a measure of the growing season and water availability (Berg et al. 2017), appeared to be the most frequent factor correlated with GS in our dataset. However, we did not observe consistent relationship between GS variation and geography, either between taxa or within taxa across ploidy levels, consistent with patterns previously observed in *A. oleraceum* (Duchoslav et al. 2013). The intraspecific intra-ploidy GS variation observed in the studied *Allium* taxa appears to be complex and idiosyncratic to each taxon. This likely reflects phylogenetic heterogeneity (Rešetnik et al. 2014), as well as historic processes associated with range shifts during the Holocene or survival in refugia where several lineages may have evolved in isolation (Nieto Feliner 2014; Horák et al. 2023). Therefore, both selection and genetic drift may have influenced the evolution of divergent GS in topographically and ecologically complex landscapes of regions like the Balkan Peninsula (Griffiths et al. 2004; Španiel and Rešetnik 2022). Similar results were previously obtained, for example, by Frajman et al. (2015) for representatives of *Knautia* L. and Terlević et al. (2022) for *Dianthus sylvestris* Wulfen. Longitudinal gradients in GS observed within some widely distributed *Allium* taxa might also reflect westward migration in the past, as discussed above. Future studies should focus on these taxa to ascertain whether there is a strong correlation between intraspecific GS variation and phylogeny, and to determine whether any taxonomic conclusions can be drawn.

Similarly, but at the interspecific level, Wang et al. (2023) found no significant relationship between GS and 19 bioclimatic variables in the 62 *Allium* species from the Qinghai-Tibetan Plateau in China. Also, Veselý et al. (2012), analysing 219 European geophytic species, found no relationship between GS and Pignatti's indicators of continentality, moisture and temperature, with only a tendency for species with very high GS to avoid water-stressed environments. Indeed, in the *Allium* species we studied, taxa with very high GS (2C > 40 pg, polyploids only) usually inhabit less stressful, often man-made habitats with higher nutrient availability (i.e., arable fields, vineyards, road ditches, moist grasslands, secondary forests; e.g., Brullo et al. 2008; Duchoslav et al. 2020, see discussion above), representing new niches for *Allium* polyploids (Hanelt 1996).

### GS as an extra taxonomic character for species identification?

FCM has repeatedly proven to be a valuable tool in biosystematic research (e.g., Zonneveld 2001; Castro et al. 2012; Lepší et al. 2019; Popelka et al. 2019; Kobrlová et al. 2022), and GS is considered an efficient tool for taxa discrimination, especially in morphologically challenging groups (e.g., Prančl et al. 2018; Sochor et al. 2019). In our study of *A*. sect. *Codonoprasum*, we found GS, when combined with classical karyology, to be useful despite the observed intraspecific GS variation in some species. An illustrative example could be the morphologically similar triplet *A. tenuiflorum/A. dentiferum*/*A. pallens*, where *A. tenuiflorum* is the predominantly diploid and the rest are exclusive polyploids, i.e., *A. dentiferum*: 4*x*, 5*x* and *A. pallens*: 4*x*. Despite the presence of the same ploidy level (4*x*), the two latter species differ in AGS and RGS, with no overlap (Table 1, Fig. 4A). In our pilot screening, we also detected a difference in GS between diploid cytotypes of two often barely distinguishable related species occurring in the same area, *A. karsianum* and *A. kunthianum* (Table 1). Moreover, FCM was also effective in distinguishing between closely related and morphologically similar species of the *A. paniculatum* complex (Fig. 4A, Table 1, see Vojtěchová et al. 2023 for details). Furthermore, FCM could also be potentially promising for the revision of the *A. stamineum* complex (sensu Brullo et al. 2007), as members measured by FCM differ in 1C*x* values. As precise genetic identification is required for further taxonomic assessment of such complexes, FCM may facilitate their delimitation.

## Conclusion

This study showed the advantage of FCM for ploidy screening and estimation of GS in the *A*. sect. *Codonoprasum*. Genome size should be regularly used as an additional taxonomic character to differentiate closely related taxa, to detect and/or resolve taxonomic heterogeneity within species groups in the genus *Allium*, and to reduce misidentifications, particularly of poorly developed individuals (Salmeri et al. 2016; Vojtěchová et al. 2023). However, our population-based FCM data also clearly underscore the need for comprehensive geographic sampling. This will provide valuable insights for future phylogenetic assessments of the taxonomically complex *A*. sect. *Codonoprasum*.

## Supplementary Information

Additional file 1.

Additional file 2.

## Data Availability

All data generated or analysed during this study are included in this published article [and its supplementary information files].
