# Supplementary material for: New estimates and synthesis of chromosome numbers, ploidy levels and genome size variation in Allium sect. Codonoprasum: advancing our understanding of the unresolved diversification and evolution of this section
Source: Bot Stud. 2024 Dec 24;65:40. doi: 10.1186/s40529-024-00446-8 (PMC11668721; doi:10.1186/s40529-024-00446-8)

**Supplementary Figure S1.** Mitotic metaphase chromosomes of the studied taxa of *A.* sect. *Codonoprasum*. **A.** *A. aetnense* (2n = 2*x* = 16, population ID 22/58, Sicily, Passopisciaro); **B.** *A. carinatum* subsp. *carinatum* (2n = 2*x* = 16, population ID 11/53, Slovakia, Velké Leváre); **C.** *A. carinatum* subsp. *carinatum* (2n = 3*x* = 24, population ID 08/12A, Slovenia, Postojna); **D.** *A. carinatum* subsp. *pulchellum* (2n = 2*x* = 16, population ID 15/48, Serbia, Nova Varoš); **E.** *A. daninianum* (2n = 2*x* = 16, population ID 23/162, Israel, Haifa); **F.** *A. dentiferum* (2n = 4*x* =32, population ID 23/01, Crete, Pano Saktouria). Bar = 20 μm. Photographs by Lucie Kobrlová.


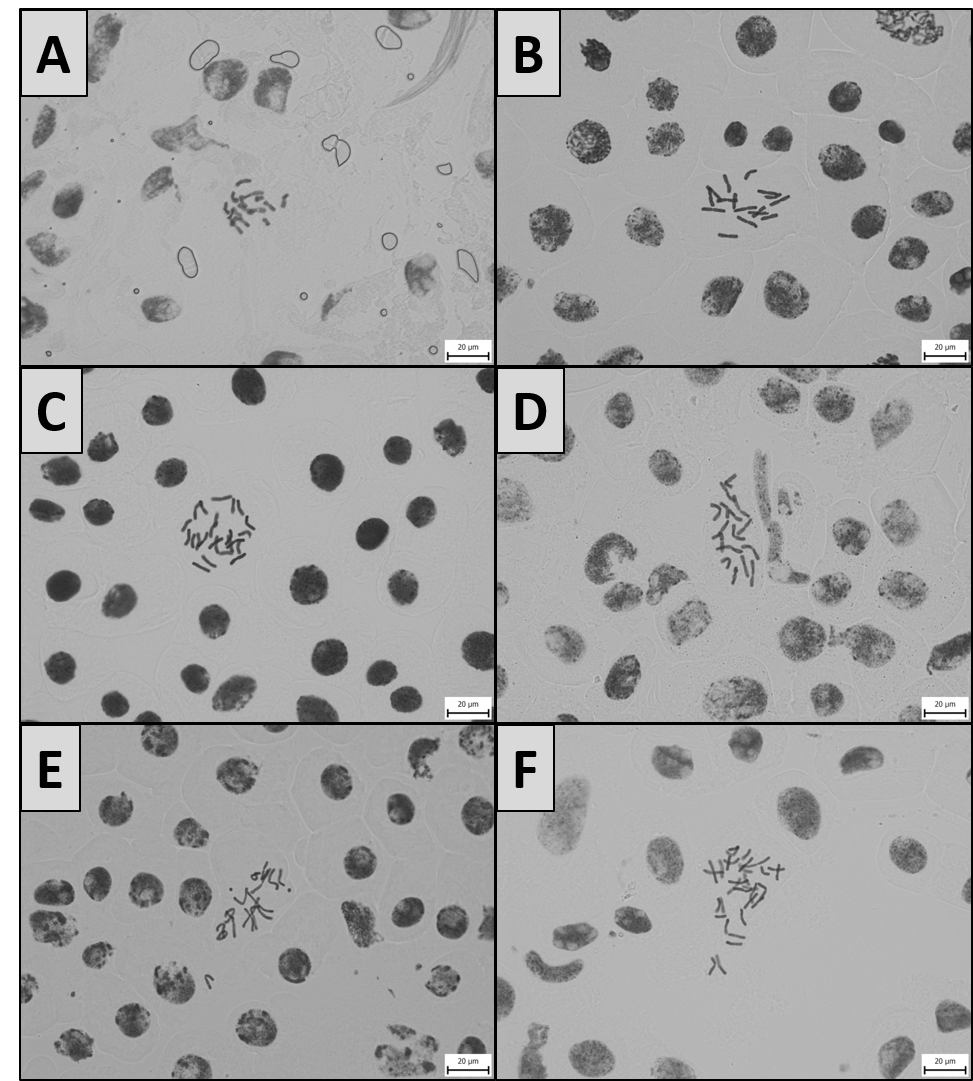


**G.** *A. dentiferum,* (2n = 5*x* = 40, population ID 10/11A, Italy, San Germano); **H.** *“A. dentiferum-pallens”* (2n = 4*x* = 32, population ID 23/179, Spain, Órgiva); **I.** *A. dinaricum* (2n = 2*x* = 18, population ID 15/46, Montenegro, Šljivansko); **J.** *A. flavum* subsp. *flavum* (2n = 2*x* = 16, population ID 04/23, Slovakia, Plešivec); **K.** *A. flavum* subsp. *flavum* (2n = 4*x* = 32, population ID 14/101, Bulgari, Kokayane); **L.** *A. flavum* subsp. *tauricum* (2n = 4*x* = 32, population ID 15/308, Ukraine, Novokairy). Bar = 20 μm. Photographs by Lucie Kobrlová and Alena Fišerová.


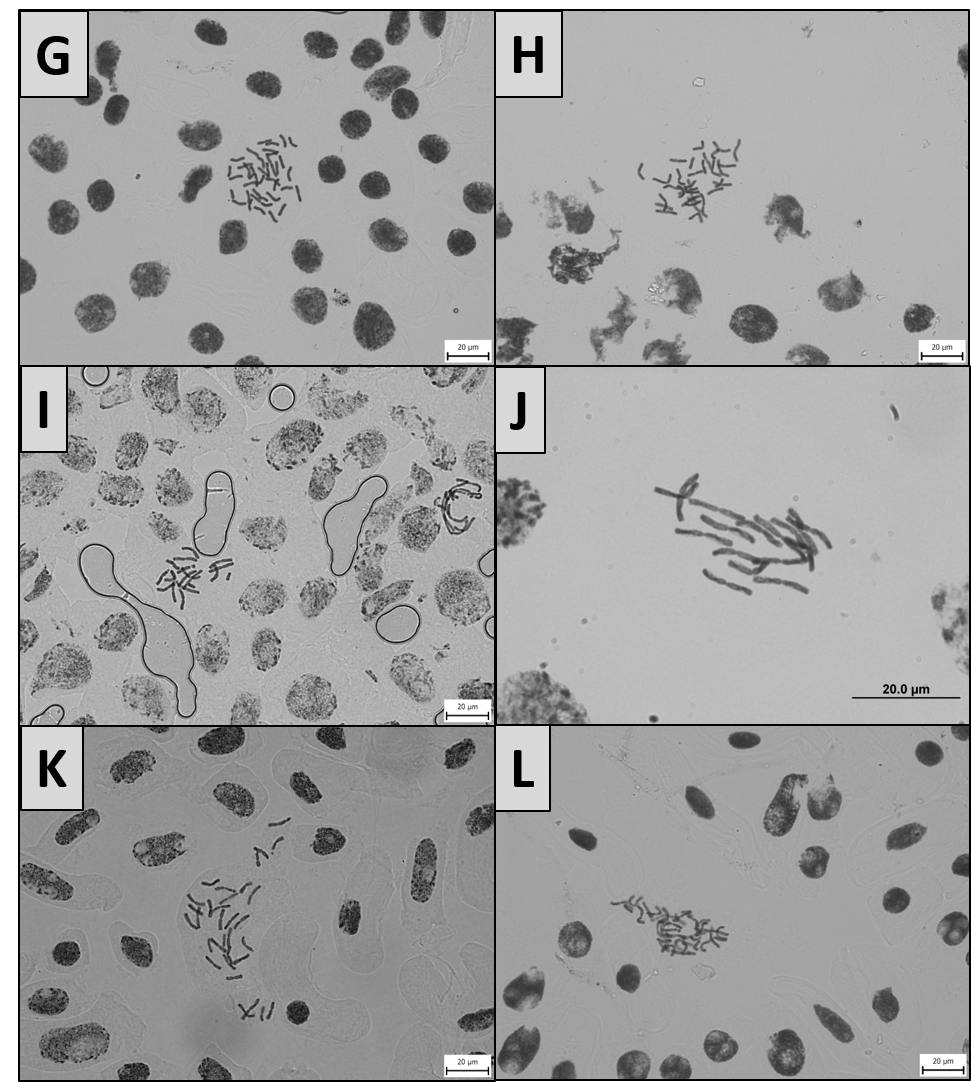


**M.** *A. garbarii* (2n = 2*x* = 16, population ID 22/04, Italy, Crotone); **N.** *A. hermoneum* (2n = 4*x* = 32, population ID 23/171, Israel, Majdal Shams); **O.** *A. karsianum* (2n = 2*x* = 16, population ID 19/117, Georgia, Ušguli); **P.** *A. kunthianum* (2n = 2*x* = 16, population ID 23/137, Georgia, Samcche-Džavachetie); **Q.** *A. melanantherum* (2n = 3*x* = 24, population ID 14/56A, Bulgaria, Rilski Manastir); **R.** *A. oporinanthum* (2n = 4*x* = 32, population ID 12/62B, Spain, La Roca del Vallès). Bar = 20 μm. Photographs by Lucie Kobrlová.


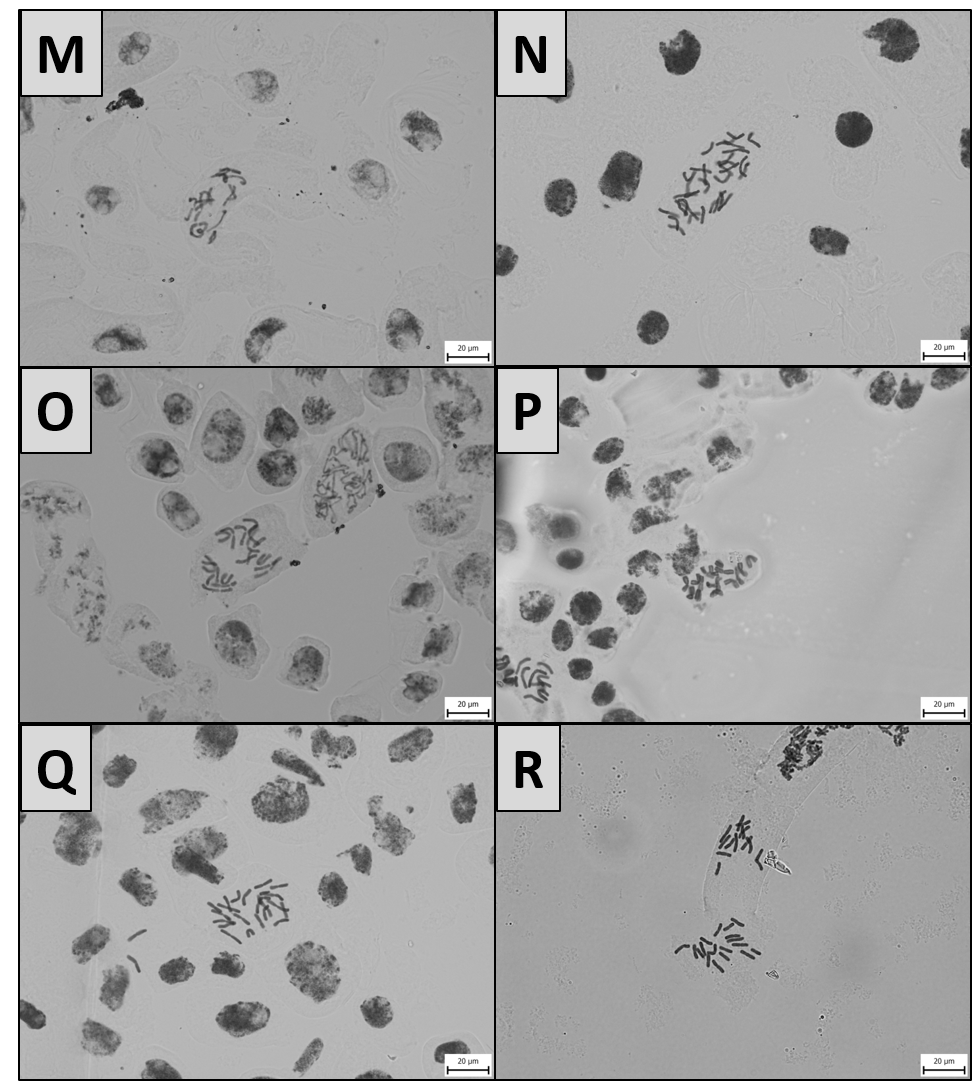


**S.** *A. pallens* (2n = 4*x* = 32, population ID 22/40, Italy, Altamura); **T.** *A. rhodopeum* (2n = 2*x* = 16, population ID 17/134, Greece, Thiva); **U.** *A. rupestre* (2n = 2*x* = 14 (*x* = 7), population ID 13/60, Georgia, Chvabiani); **V.** *A. rupestre* (2n = 3*x* = 21 (*x* = 7), population ID 12/04A, Ukraine, Nikita); **W.** *A. rupestre* (2n = 4*x* = 28 (*x* = 7), population ID 13/65, Georgia, Kvabiskhevi); **X.** *A. rupestre* (2n = 3*x* = 24 (*x* = 8), population ID 18/32, Georgia, Didgoori). Bar = 20 μm. Photographs by Lucie Kobrlová and Michaela Jandová.


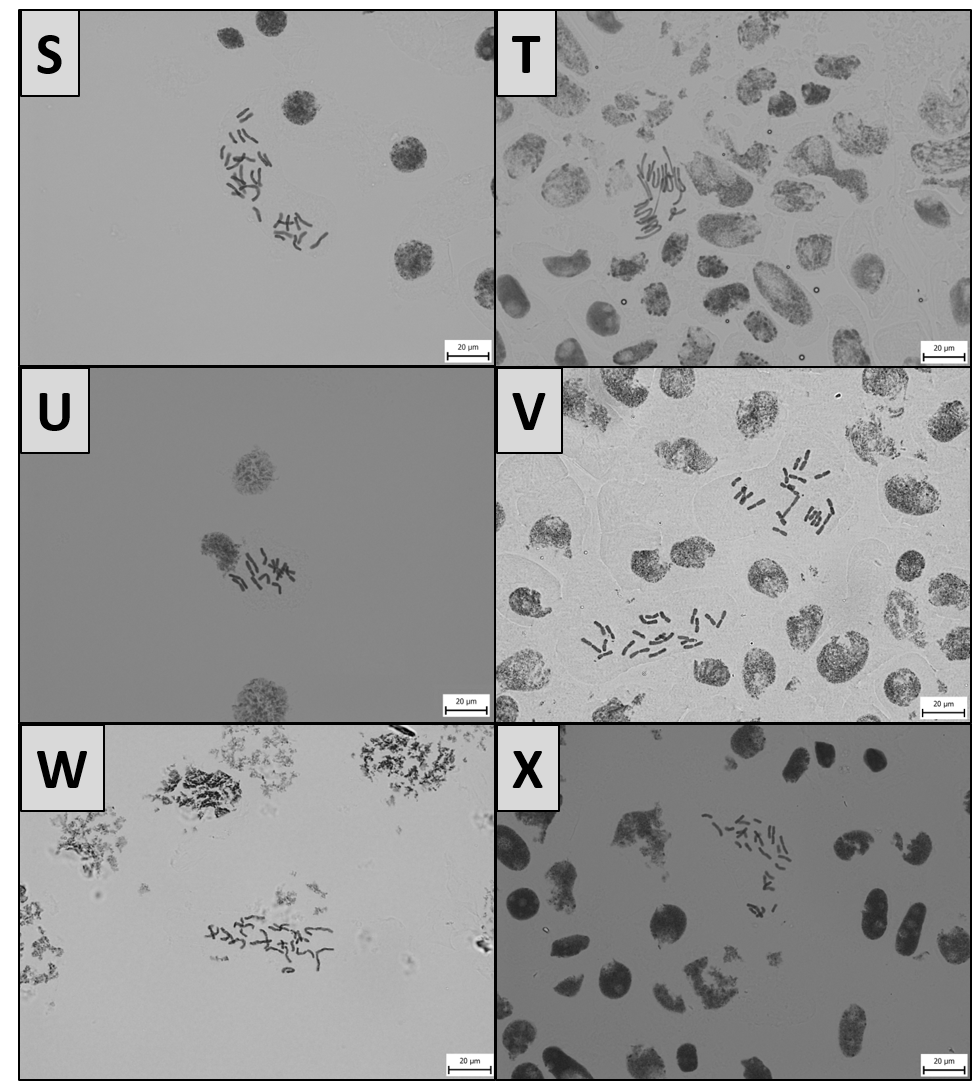


**Y.** *A. telmatum* (2n = 4*x* = 32, population ID 15/42B, Croatia, Nin); **Z.** *A. tenuiflorum* (2n = 2*x* = 16, population ID 23/190B, Croatia, Pula, Verudela); **AZ.** *A. valdesianum* (2n = 2*x* = 16, population ID 23/175, Spain, Monachil). Bar = 20 μm. Photographs by Lucie Kobrlová.


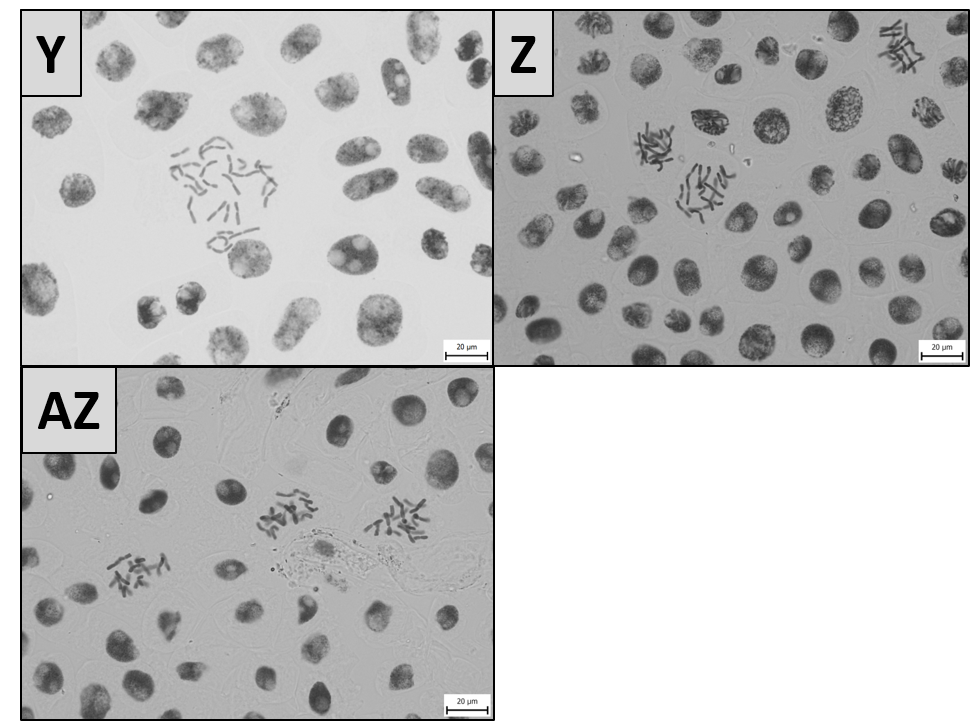


**Supplementary Figures S2.** Variation in RGS for taxa of *A.* sect. *Codonoprasum* with a wider range of distribution. Circle sizes are proportional to RGS values within each taxon only. The original RGS values for particular ploidies/taxa used in the maps are given in Table S1. The approximate range of a given taxon is shown in light yellow. *Allium carinatum* subsp. *carinatum* (2n = 2*x*), *Allium carinatum* subsp. *carinatum* (2n = 3*x*), *Allium carinatum* subsp. *carinatum* (2n = 4*x*), *Allium carinatum* subsp. *pulchellum* (2n = 2*x*), *Allium dentiferum* (2n = 4*x*), *Allium dentiferum* (2n = 5*x*), *Allium “dentiferum-pallens”* (2n = 4*x*), *Allium pallens* (2n = 4*x*), *Allium flavum* subsp. *flavum* (2n = 2*x*), *Allium flavum* subsp. *flavum* (2n = 4*x*), *Allium flavum* subsp. *tauricum* (2n = 2*x*), *Allium flavum* subsp. *tauricum* (2n = 4x).


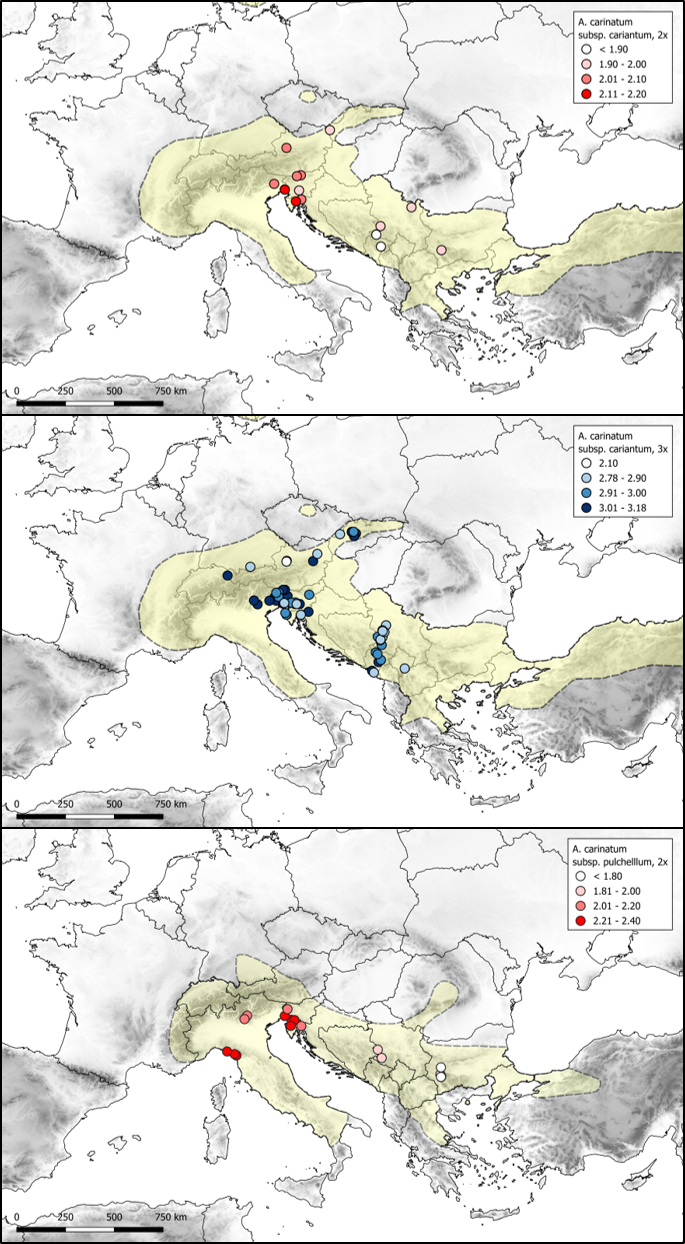


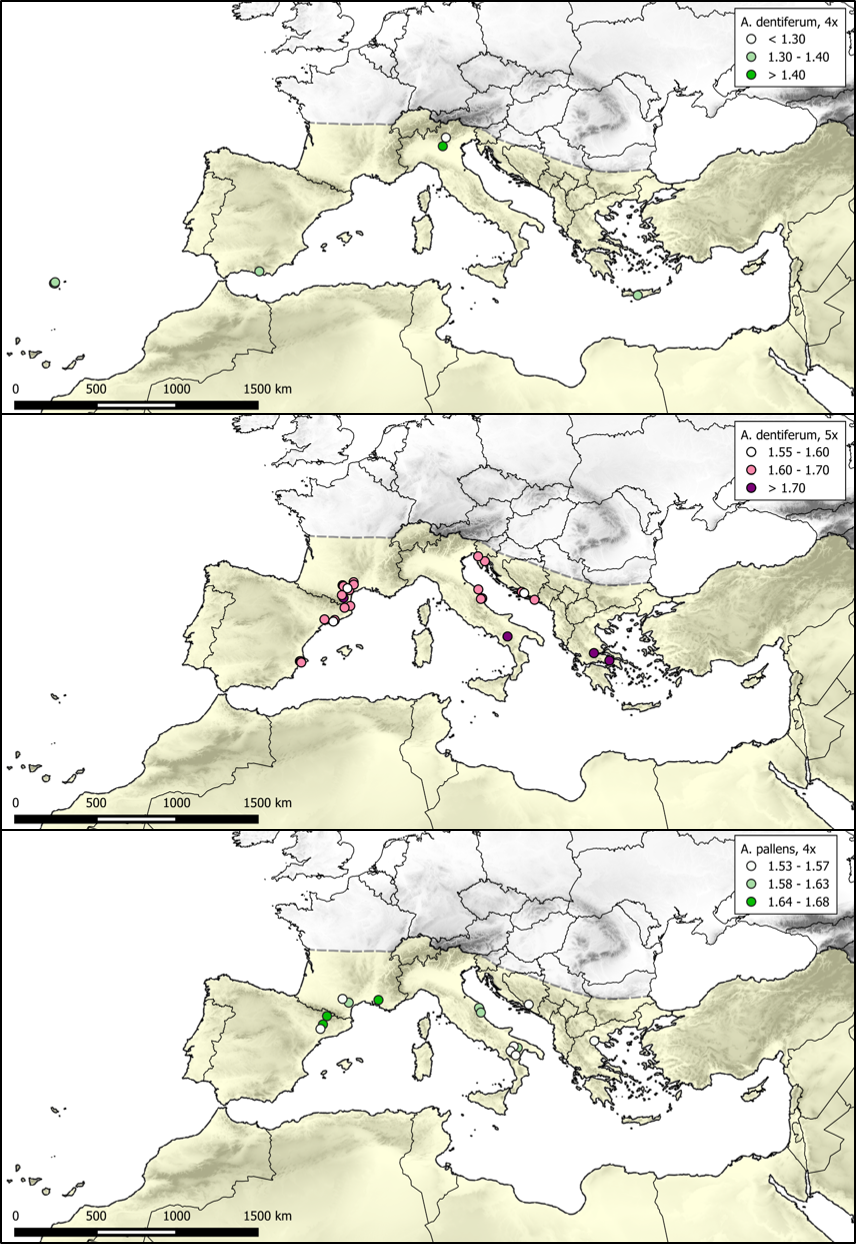


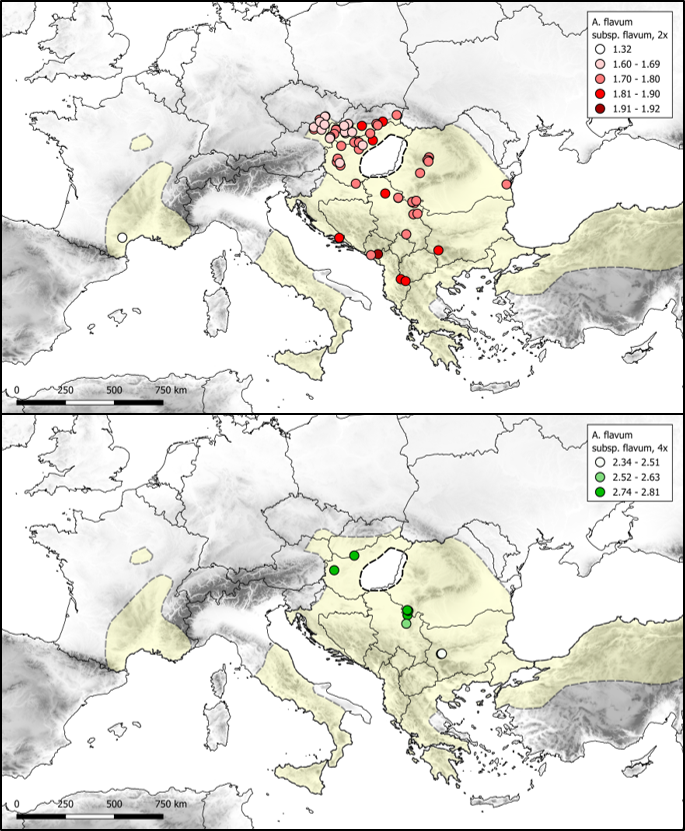


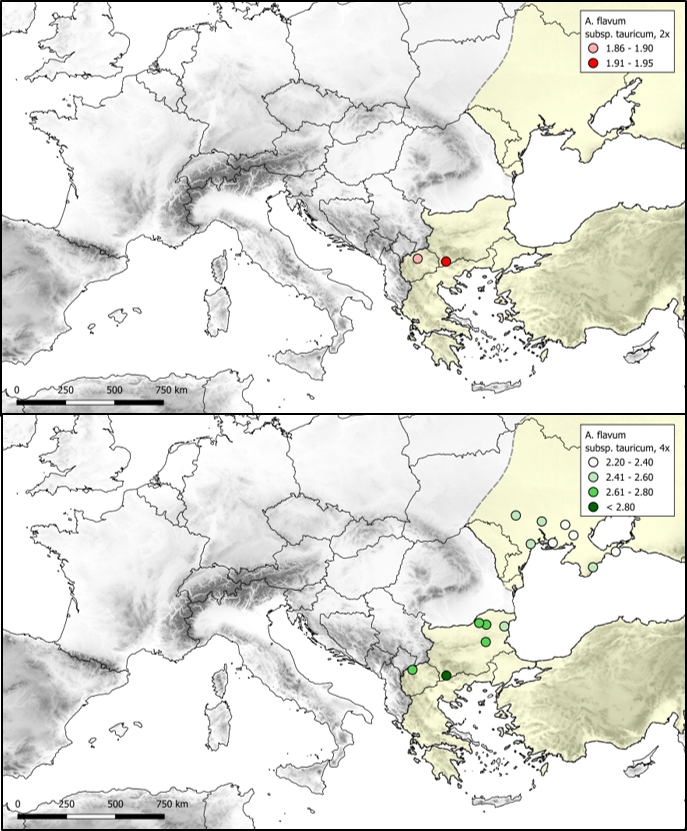

Supplement: Supplementary file 2 — Additional file 2. [file 40529_2024_446_MOESM2_ESM.docx]
